# Synergistic combination of RAD51-SCR7 improves CRISPR-Cas9 genome editing efficiency by preventing R-loop accumulation

**DOI:** 10.1016/j.omtn.2024.102274

**Published:** 2024-07-17

**Authors:** Sun-Ji Park, Seo Jung Park, Yang Woo Kwon, Eui-Hwan Choi

**Affiliations:** 1 New Drug Development Center, Daegu-Gyeongbuk Medical Innovation Foundation (DGMIF), Deagu 41061, South Korea; 2 New Drug Development Center, Osong Medical Innovation Foundation, Cheongju 28160, South Korea

**Keywords:** MT: RNA/DNA editing, CRISPR-Cas9, RAD51, SCR7, homologous recombination, DNA repair, R-loop

## Abstract

CRISPR-Cas9 has emerged as a powerful tool for genome editing. However, Cas9 genome editing faces challenges, including low efficiency and off-target effects. Here, we report that combined treatment with RAD51, a key factor in homologous recombination, and SCR7, a DNA ligase IV small-molecule inhibitor, enhances CRISPR-Cas9-mediated genome-editing efficiency in human embryonic kidney 293T and human induced pluripotent stem cells, as confirmed by cyro- transmission electron microscopy and functional analyses. First, our findings reveal the crucial role of RAD51 in homologous recombination (HR)-mediated DNA repair process. Elevated levels of exogenous RAD51 promote a post-replication step via single-strand DNA gap repair process, ensuring the completion of DNA replication. Second, using the all-in-one CRISPR-Cas9-RAD51 system, highly expressed RAD51 improved the multiple endogenous gene knockin/knockout efficiency and insertion/deletion (InDel) mutation by activating the HR-based repair pathway in concert with SCR7. Sanger sequencing shows distinct outcomes for RAD51-SCR7 in the ratio of InDel mutations in multiple genome sites. Third, RAD51-SCR7 combination can induce efficient R-loop resolution and DNA repair by enhanced HR process, which leads to DNA replication stalling and thus is advantageous to CRISPR-Cas9-based stable genome editing. Our study suggests promising applications in genome editing by enhancing CRISPR-Cas9 efficiency through RAD51 and SCR7, offering potential advancements in biotechnology and therapeutics.

## Graphical abstract

**Figure:**
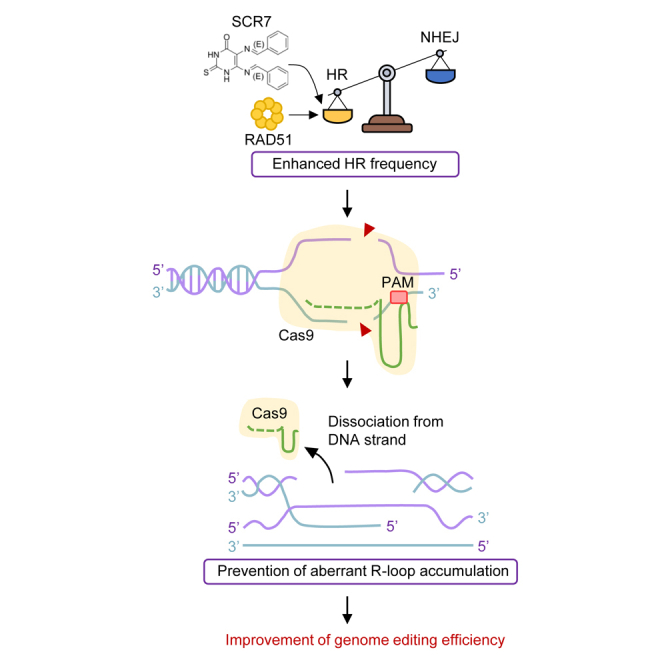

## Teaser

Choi and colleagues reveal a synergistic strategy employing RAD51 and SCR7 to enhance HDR, significantly improving genome-editing efficiency. This innovative approach prevents R-loop accumulation, offering valuable insights into precise genome editing. The findings hold promise for advancing therapeutic applications and addressing limitations in current CRISPR-Cas9.

## Introduction

In clustered regularly interspaced short palindromic repeat (CRISPR)-regulated genome editing, CRISPR-associated protein (Cas9) endonucleases generate double-strand breaks (DSBs) at target sites complementary to the guide RNA (gRNA) spacer sequence.^1^^,^^2^^,^^3^ Cas9 initially recognizes a specific protospacer adjacent motif (PAM) sequence next to the target site, triggering DNA double-strand separation to initiate directional hybridization with gRNA, forming an RNA-DNA hybrid known as the R-loop structure. The formation of a complete R-loop leads to the conformational activation of the Cas9 nuclease domains to generate DSBs.^3^^,^^4^ Cellular repair of these DSBs can result in the precise introduction of genetic mutations, while inadequate repair leads to cell death.^5^^,^^6^ DSBs are repaired through homologous recombination (HR) or non-homologous end-joining (NHEJ).^5^^,^^7^ Error-prone NHEJ is a homologous template-independent pathway that repairs by directly re-ligating broken DNA ends. NHEJ-based DSB repair is particularly useful in genetic knockout experiments by inducing insertions/deletions (InDels) mutations at the DSB site, but NHEJ is inaccurate and often leads to unintended mutations.^8^^,^^9^ In contrast, the error-free HR pathway facilitates experimentally targeted genome modifications using sister chromatids as templates. Although HR has a low frequency and is a relatively complex process compared to the NHEJ pathway, it serves as a superior genome engineering strategy owing to its high fidelity and precise editing.^5^^,^^8^^,^^10^ However, Cas9-mediated gene editing has limitations, including low efficiency and off-target effects that occur when Cas9 acts on an untargeted genomic site.

Recent studies emphasize enhancing genome-editing efficiency by suppressing the NHEJ pathway through inhibition of main enzymes, such as DNA ligase IV, Ku heterodimer (Ku80/70), and DNA-dependent protein kinases (DNA-PKcs), or by facilitating the HR pathway using a single-stranded oligodeoxynucleotide template containing homology arms and the desired edit sequence.^10^^,^^11^^,^^12^

RAD51, a crucial strand exchange repair protein, plays an essential role in the HR pathway. In response to DSB, the single-stranded DNA (ssDNA) binding protein replication protein A (RPA) coats 3′ terminated ssDNA overhangs resulting from end resection. RAD51 can replace RPA and bind to the ssDNA overhangs, which forms long helical nucleofilaments.^13^^,^^14^^,^^15^ RAD51 nucleofilaments actively perform the search for homologous sequences of the donor DNA template and strand invasion of the 3′ ssDNA overhangs into the homologous dsDNA.^15^^,^^16^ Therefore, this step is absolutely required in modulating HR.^17^^,^^18^ Since RAD51-dependent HR is one of the most important steps in HR-mediated DNA repair, it has been assumed that RAD51 has the capacity to improve HR efficiency.

Our previous study demonstrated that elevated levels of exogenous RAD51 significantly improve CRISPR-Cas9-mediated gene knockin and knockout efficiency.^18^ In this study, we investigated the underlying mechanism through which RAD51 enhances HR efficiency. To promote genome-editing efficiency, we attempted to increase HR efficiency using SCR7 and by increasing the expression of exogenous RAD51 using the all-in-one CRISPR-Cas9-RAD51 system. SCR7 acts as an inhibitor of DNA ligase IV, a key enzyme in the NHEJ pathway, by directly binding to DNA binding domain and disrupting the progression of NHEJ events. NHEJ inhibition by SCR7 has been shown to increase the efficiency of HR-mediated genome editing using CRISPR-Cas9 in mammalian cells and mouse embryos. However, the role of combined RAD51 and SCR7 in inducing HR remains controversial.^19^^,^^20^ Our findings indicate that both RAD51 overexpression and NHEJ inhibition by SCR7 increased HR repair by 7.75% in Human embryonic kidney (HEK) 293T cells. Notably, enhancement of the HR pathway not only increased knockin and knockout efficiency by over 70% with gRNA targeting GAPDH or ATM but also increased the InDel mutation in stable HEK293T cells and human induced pluripotent stem cells (hiPSCs) expressing Cas9-gRNA. Analysis of R-loop formation revealed that the combined treatment with RAD51 and SCR7 prevented R-loop accumulation by activating HR-based repair. Our results suggest a novel mechanism that the combined RAD51 and SCR7 maintain R-loop stability and prevent R-loop accumulation by facilitating efficient HR-induced DSB repair, ultimately leading to enhanced CRISPR-Cas9 genome-editing efficiency.

## Results

### RAD51 knockdown induces cell death by reducing HR-related repair efficiency

**Figure 1:**
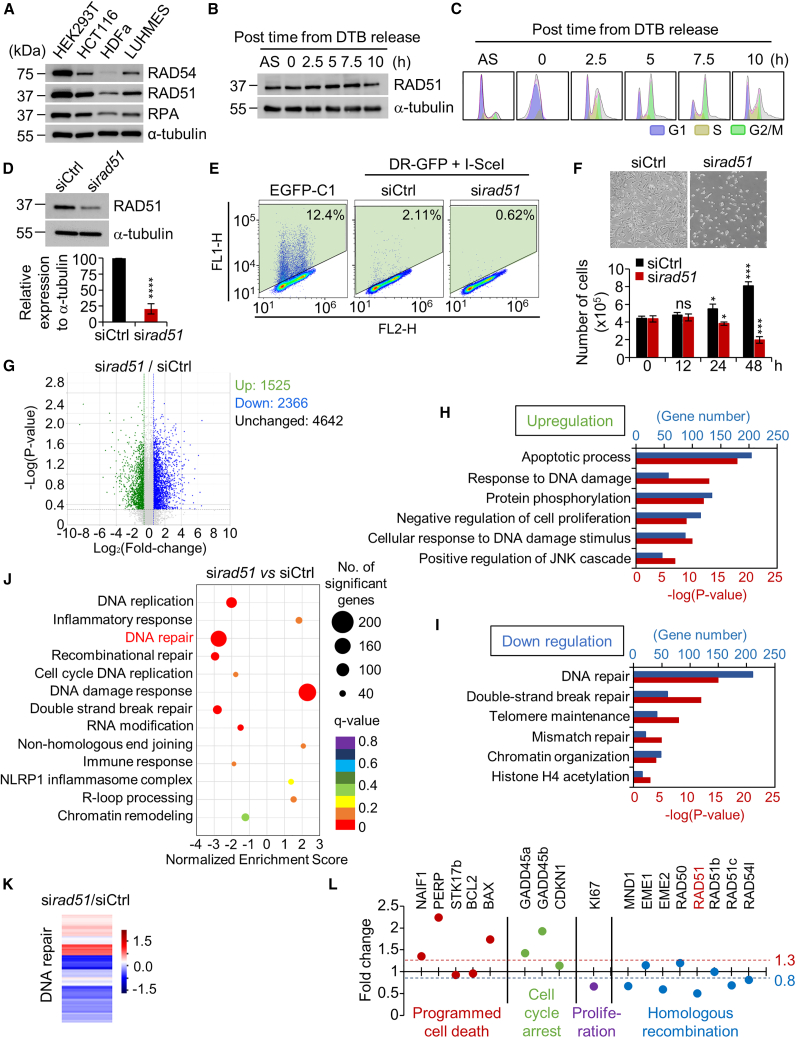
Alterations in cell proliferation, DNA repair ,and gene expression pattern in response to RAD51 knockdown (A) Comparison of the expression levels of HR-related proteins in four cell types: HEK293T (human embryonic kidney), HCT116 (human colorectal cancer), HDFa (human dermal fibroblast adult), and LUHMES (Lund human mesencephalic cell). (B) The expression pattern of RAD51 after time-dependent experiment performed on HEK293T cells. DTB, double thymidine block. (C) FACS analysis showing cell-cycle pattern following G1-phase synchronization in HEK293T cells. The cell-cycle intervals are 2.5 h. AS, asynchronous state. (D) Comparison of RAD51 knockdown efficiency by siRNA pool against RAD51. The level of RAD51 protein was normalized to the α-tubulin (bottom). (E) Analysis of HR repair assay. HEK293T pDR-GFP cells were transfected with pCBA-SceI plasmid and then analyzed by flow cytometry. pEGPF-C1 plasmid was used as a GFP-positive control. FITC signal (FL1), PE signal (FL2). (F) Representative phase-contrast image of HEK293T cells at 48 h following transfection with control siRNA or si*rad51*. The total number of cells were quantified in control cells and RAD51 knockdown-induced cells at the indicated times after transfection (bottom). Quantification data represent the mean ± SD of three independent experiments. *p* values (paired two-tailed t test) were analyzed with GraphPad Prism 9 software. ∗*p* < 0.05; ∗∗∗*p* < 0.001; ns, not significant. (G) The volcano plot of 1,525 upregulated, 2,366 downregulated, and 4,642 unchanged genes. The volcano plots of si*rad51*/siCtrl were overlapped in one plot, and commonly regulated gene sets are indicated with colors. Green, upregulation with log_2_(fold change) < −0.5, −log(*p* value) > 0.3; Blue, downregulation with log_2_(fold change) < 0.5, −log(*p* value) > 0.3. (H and I) Analyses of the DEGs were performed by using the DAVID database. Enriched biological themes, especially GO terms, were identified in both upregulated and downregulated genes. Blue bar indicates the number of up-/downregulated genes in each cluster. Red bar indicates −log(*p* value) in each cluster. (J) GSEA score of si*rad51* HEK293T cells versus siCtrl HEK293T cells. GSEA data were analyzed with total RNA sequencing data from two independent experiments. (K) Heatmap from GSEA of DNA repair-related pathway genes in si*rad51* HEK293T cells versus siCtrl HEK293T cells. Red and blue bars indicate upregulation and downregulation, respectively. (L) The transcription level of programmed cell death, cell-cycle arrest, proliferation, and HR-regulated genes in si*rad51* HEK293T cells. Y axis indicates the average fold change from two independent RNA sequencing data. Red and blue lines represent the upregulation (fold change >1.3) and downregulation (fold change <0.8), respectively.

CRISPR-Cas9 generates DSBs, initiating cellular DNA repair processes crucial for genome editing. Efficient DNA repair is an essential step in the genome-editing process and maintenance of cellular proliferation.^21^^,^^22^ To determine whether RAD51, the main factor in HR-related DNA repair, regulates both replication and genome-editing processes, we examined the expression levels of RAD51 and HR-related proteins in four cell types: HEK293T, HCT116, HDFa, and lund human mesencephalic (LUHMES). Expression levels of RAD51, RAD54, and RPA were notably higher in HEK293T cells than in all other types (Figure 1A). RAD51 expression patterns were further characterized in asynchronous HEK293T cells using a double thymidine block (DTB). HEK293T cells exhibited constitutive RAD51 expression independent of the cell cycle (Figures 1B and 1C). Due to their consistently high RAD51 expression, HEK293T cells were selected for this study. To directly test the role of RAD51 in the regulation of cell viability, we used small interfering RNA (siRNA) to suppress RAD51 expression in HEK293T cells. RAD51 expression was reduced by 79.5% in cells transfected with siRNA against RAD51 (si*rad51*) compared to that in cells transfected with control siRNA (siCtrl) (Figure 1D). We also performed a direct repeat green fluorescent protein (DR-GFP) assay to evaluate HR repair activity.^23^^,^^24^ si*rad51* cells were unable to stimulate HR activity to the same extent as siCtrl cells, indicating that RAD51 knockdown suppressed the HR pathway (Figure 1E). Furthermore, the viability of si*rad51* cells significantly decreased at 24 h (30.6%) and 48 h (75.5%)*,* whereas siCtrl cell viability continued to increase post transfection (Figure 1F). These results underscore the critical role of high RAD51 levels in maintaining HEK293T cell proliferation through an efficient HR repair pathway.

### RAD51 depletion inhibits the expression of DNA repair and cell proliferation-regulated genes

To analyze the differences in genome-wide HR-related gene expression between siCtrl and si*rad51* cells, total RNA sequencing was performed. Volcano plots illustrated 1,525 upregulated, 2,366 downregulated, and 4,642 unchanged genes, offering insights into RAD51 regulation patterns (Figure 1G). Analysis of differentially expressed genes (DEGs) identified enriched pathways using Gene Ontology (GO) functional analysis and DAVID functional annotation bioinformatics. The top six enriched annotation clusters of upregulated genes comprised those associated with apoptosis, DNA damage response, protein phosphorylation, negative regulation of cell proliferation, cellular response to DNA damage stimuli, and positive regulation of the JNK cascade (Figure 1H). The leading six annotation clusters of downregulated genes aligned with functions related to DNA repair, DSB repair, telomere maintenance, mismatch repair, chromatin organization, and histone H4 acetylation (Figure 1I). Furthermore, gene set enrichment analysis (GSEA) revealed a significant decrease in the expression levels of genes related to DNA repair, recombination repair, and DSB repair in si*rad51* cells compared to siCtrl-transfected cells (Figure 1J). In addition, the DNA repair pathway exhibited a higher ratio of downregulated genes in si*rad51* cells than in siCtrl cells (Figure 1K).

We further evaluated the altered transcriptional levels of DNA repair pathway-associated genes in response to RAD51 knockdown. RNA-seq analysis showed that RAD51 inhibition regulated many genes involved in programmed cell death, cell-cycle arrest, proliferation, and HR (Figure 1L). The transcription levels of the proliferation marker KI67 and HR proteins MND1, EME2, RAD51, and RAD54 were lower in si*rad51* cells than in siCtrl cells. In contrast, the expression levels of PERP and BAX, known apoptosis regulators, were markedly increased by 2.24-fold and 1.74-fold, respectively, in si*rad51* cells (Figure 1L). Notably, GADD45a and GADD45b, pivotal players in the G2/M cell-cycle checkpoint, were strongly upregulated by RAD51 knockdown (Figure 1L). Flow cytometric analysis showed that si*rad51* cells exhibited an increase in the proportion of G2/M phase cells and a decrease in the proportion of G0/G1 phase cells, indicating that RAD51 knockdown induced G2/M arrest by upregulating the transcription of GADD45a and GADD45b (Figure S1). Collectively, these data suggest that the impaired DNA repair pathway induced by RAD51 inhibition was associated with cell-cycle arrest in the G2/M phase, suppressing cell proliferation and leading to apoptotic cell death.

### RAD51 inhibition collapses HR-mediated DSB repair system

**Figure 2:**
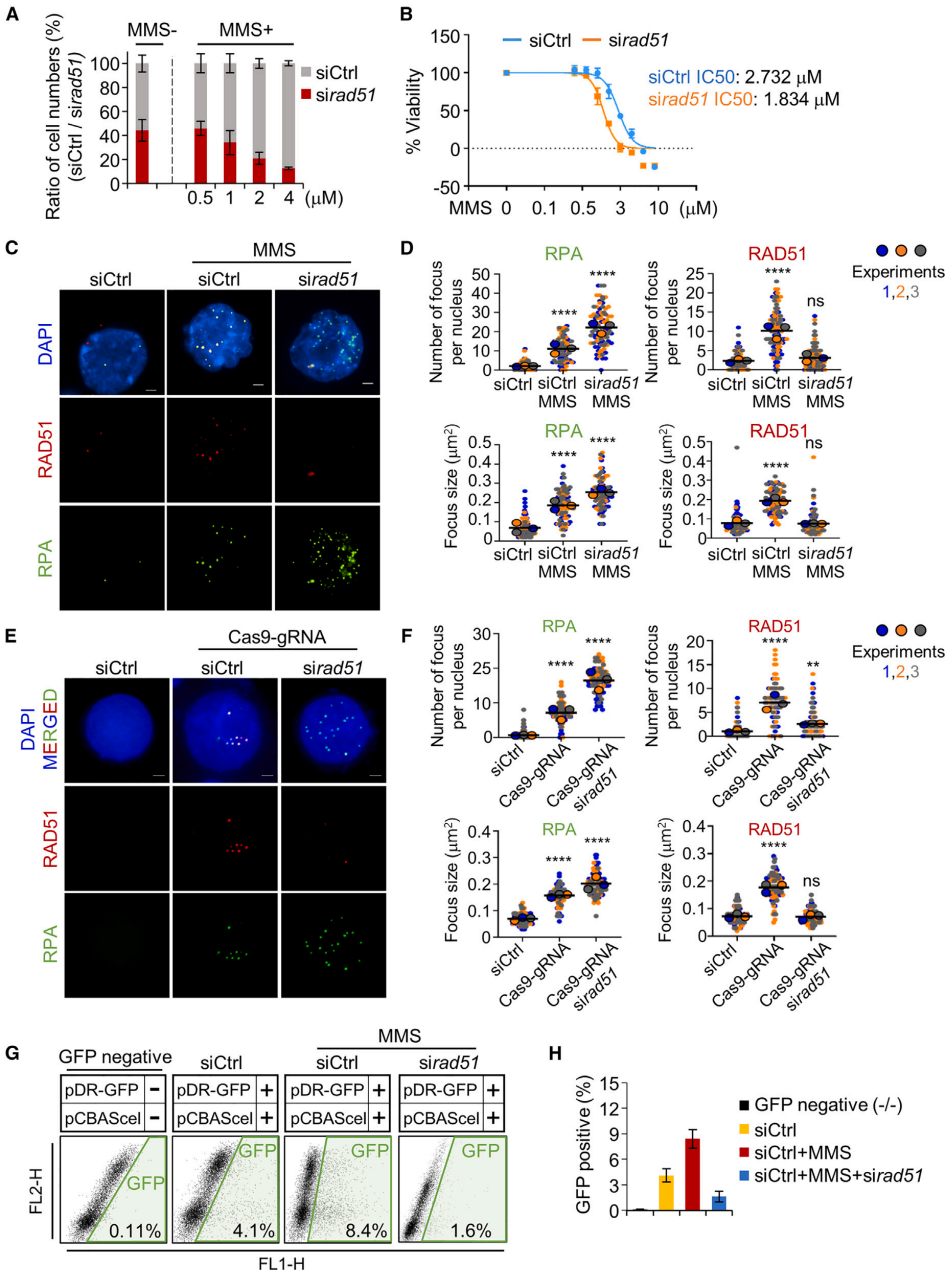
Regulation of repair process by RAD51 under DNA damage condition (A) Analysis of cell death in HEK293T cells. RAD51 was inhibited by siRNA pool against RAD51 (si*rad51*). MMS was added to HEK293T cells transfected with siCtrl or si*rad51* and incubated for 3 h. The cells were stained with (1×) trypan blue and analyzed by automatic cell counter. Three independent experiments were performed. Error bars denote the mean ± SD (*n* = 3). (B) HEK293T cell viability was calculated in response to MMS at different doses. CCK-8 analysis was performed after 3 h MMS treatment. siCtrl (IC50 = 2.732 μM) and si*rad51* (IC_50_ = 1.834 μM). Data are represented as mean ± SD (*n* = 3). (C) Representative images of RPA and RAD51 focus formation in HEK293T cells. Cells were imaged after transfection with siCtrl or si*rad51* for 48 h. Cells were treated with 1 μM MMS. Scale bars represent 2.5 μm. (D) Quantitative analysis of focus formation in response to si*rad51*. The numbers of foci and size in each condition were quantified. Super-plot is color-coded (blue, orange, gray), and the average of replicated value is represented with a larger dot, and larger bar represent the averages of three means (*n* ≥ 30 for condition). Quantification data represent the mean with SD. ∗∗∗∗*p* < 0.0001; ns, not significant; paired two-tailed t test. (E) Representative images of RPA and RAD51 focus formation in HEK293T cells. Cells were imaged after transfection with siCtrl or si*rad51* for 48 h. Cells were transfected with Cas9 and gRNA targeting hGAPDH. Scale bars represent 2.5 μm. (F) Quantitative analysis of focus formation in response to si*rad51* under DSB induced by CRISPR-Cas9. Super-plot is color-coded (blue, orange, gray), and the average of replicated value is represented with a larger dot, and larger bars represent the averages of three means (*n* ≥ 30 for condition). Quantification data represent the mean with SD. ∗∗*p* < 0.01, ∗∗∗∗*p* < 0.0001; ns, not significant; paired two-tailed t test. (G) Analysis of HR repair assay. Stable HEK293T cells containing the pDR-GFP plasmid were transfected with and without pCBASceI plasmid. These cells were transfected with siCtrl or si*rad51* and then were treated with MMS for 3h. (H) The percentage of GFP-positive cells was quantified using flow cytometry in three independent experiments. Error bars denote the mean ± SD.

To determine whether RAD51 promotes the activity of the HR pathway to induce DNA repair and complete DNA replication under DNA damage conditions, HEK293T cells transfected with either siCtrl or si*rad51* were exposed to DNA-damaging agent methyl methanesulfonate (MMS) for 3 h (Figures 2A and S2A). We tested four different concentrations (0.5, 1, 2, and 4 μM) of MMS to optimize the concentration and then determined 1 μM as an optimal MMS concentration that is capable of inducing DSBs without leading significant cytotoxicity (Figure 2A). Following treatment with 1 μM MMS, the 50% inhibitory concentration (IC_50_) was evaluated using a CCK-8 assay. According to the IC_50_ values, si*rad51* cells exhibited heightened sensitivity to MMS compared to siCtrl cells after 3 h of treatment (Figure 2B). Since, the recruitment of HR factors to DNA break sites is considerably important for DNA repair via the HR pathway, we observed the focus formation containing RPA and RAD51 bound to DNA break sites or exposed ssDNA gaps in the nucleus (Figure 2C). The average number and size of RPA and RAD51 foci per nucleus were measured: 2.12/0.068 μm (siCtrl), 11.1/0.184 μm (siCtrl+MMS), and 22.16/0.253 μm (si*rad51+*MMS) for RPA; 2.32/0.077 μm (siCtrl), 10.13/0.193 μm (siCtrl+MMS), and 3.11/0.075 μm (si*rad51*+MMS) for RAD51, respectively (Figure 2D). Interestingly, when RAD51 was depleted under MMS-induced DNA damage conditions, the number and size of RPA foci were dramatically increased dramatically by 2.00-fold and 1.38-fold, respectively, compared to MMS treatment alone (Figure 2D). RAD51 knockdown resulted in the accumulation of RPA at the break sites, supporting the hypothesis that HEK293T cells require RAD51 for MMS-induced DSB repair. We further analyzed whether CRISPR-Cas9 induces direct DSBs, and we observed focus formation of RAD51 and RPA in cells expressing Cas9-gRNA (Figure 2E). The average number and size of RPA and RAD51 foci per nucleus were measured: 0.71/0.073 μm (siCtrl), 7.23/0.158 μm (Cas9-gRNA), and 16.83/0.207 μm (Cas9-gRNA +si*rad51*) for RPA; 1.12/0.075 μm (siCtrl), 7.25/0.178 μm (Cas9-gRNA), and 2.35/0.063 μm (Cas9-gRNA+si*rad*51) for RAD51, respectively (Figure 2F). To directly demonstrate DSB induction by Cas9 or MMS, we analyzed the formation of γH2AX foci. The Cas9-gRNA or MMS induced over 7.2-fold increase in γH2AX focus formation compared to siCtrl, and RAD51 inhibition resulted in a more than 13.7-fold increase in γH2AX focus formation compared to siCtrl (Figures S2B and S2C). These observation suggested that Cas9 and MMS can induce direct DSBs in HEK293T cells.

In addition, cellular HR activity was evaluated using the DR-GFP assay. As shown in Figure 2G, 4.1% of siCtrl cells were GFP-positive, and the number of GFP-positive cells increased by 8.4% after MMS treatment. However, the percentage of GFP-positive cells rapidly decreased to ∼1.6% in response to RAD51 knockdown (Figures 2G and 2H). These results indicate that RAD51 depletion under DNA damage stress can lead to the accumulation of DNA breaks, resulting from an incomplete HR pathway. Thus, RAD51 is essential for enhancing HR-mediated DNA repair. Our findings suggest that the incorporation of RAD51 improves CRISPR-Cas9-mediated genome-editing efficiency.

### Exogenous RAD51 promotes DNA breaks repair by activating the HR process

**Figure 3:**
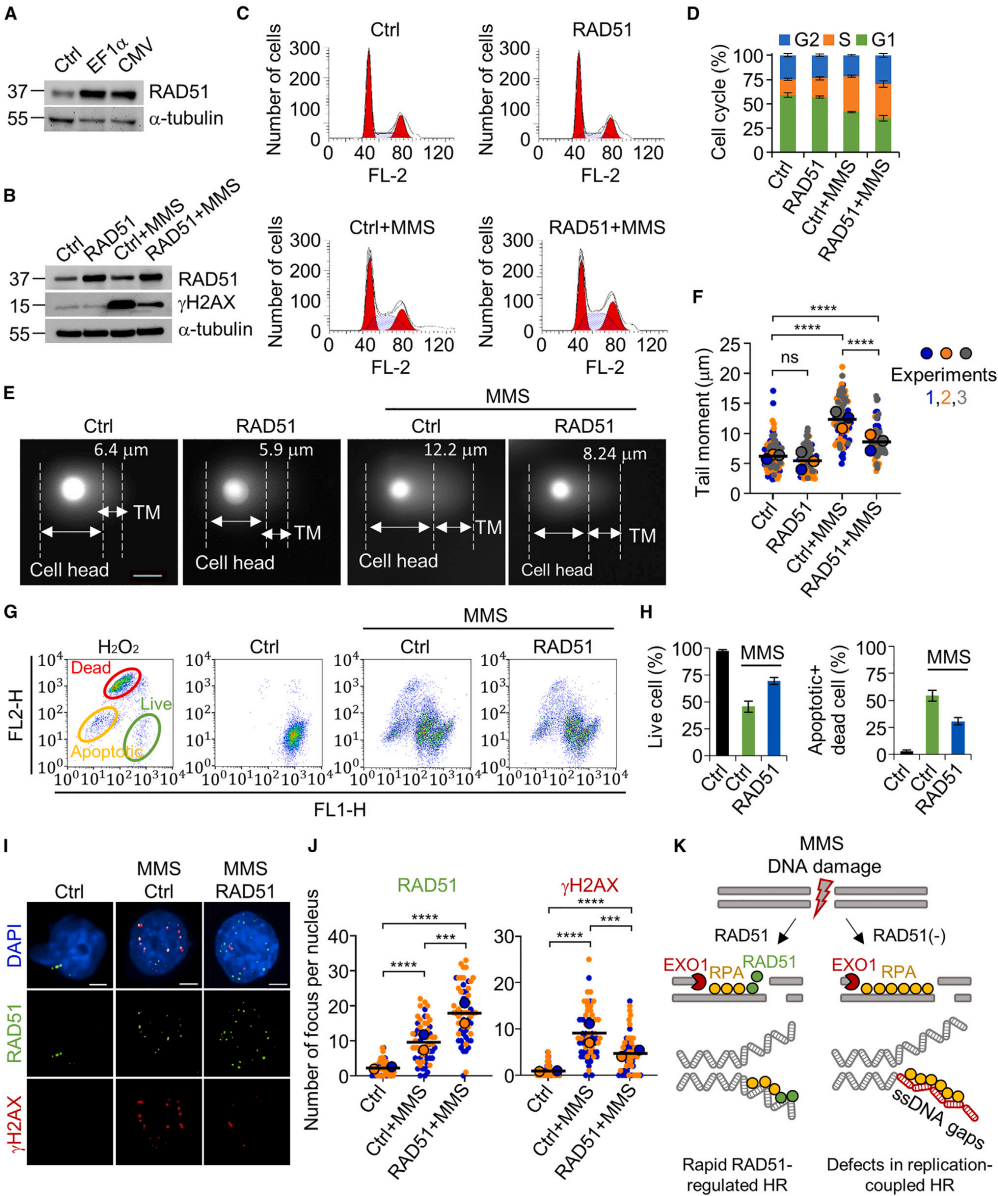
Efficiency of DNA break repair under high expression of the RAD51 (A) Comparison of RAD51 expression level between EF1α and CMV promoters. Cells transfected with an empty vector were used as a control (Ctrl) for all experiments. α-Tubulin was used as a housekeeping control. (B) Analysis of DNA damage repair under RAD51 overexpressed condition. γH2AX was used as a sensitive molecular marker of DNA damage. (C) Cell-cycle profiles of HEK293T cells. The cells in each condition were analyzed by flow cytometry and quantified with ModFit LT software. (D) Quantification of the cell-cycle ratios in (C). Error bars denote the mean ± SD (*n* = 3). (E) Calculation of the degree of DNA break using the comet assay. DNA tail moments (TM) of more than fifty independent cells per sample were analyzed with CASP software (ver. 1.2.3beta2). Scale bar represents 10 μm. (F) Calculation of DNA tail moments (tail length × percentage of DNA in the tail). Super-plot is color-coded (blue, orange, gray), and the average of replicated values is represented with a larger dot, and larger bars represent the averages of three means (*n* ≥ 30 for group). (G) Analysis of cell viability in response to DNA damage and RAD51 overexpression. Percentages of live, apoptotic, and dead cells were measured and analyzed by flow cytometry. (H) The average of live cells or apoptotic + dead cells. Error bars denote the mean ± SD (*n* = 3). (I) Representative images of RAD51 and γH2AX focus formation in HEK293T cells. Cells were imaged in siCtrl or RAD51 overexpression conditions. Cells under each condition were treated with 1 μM MMS. Scale bars represent 2.5 μm. (J) Quantitative analysis of focus formation in each condition. The numbers of foci were quantified. Super-plot is color-coded (blue, orange, gray), and the average of replicated values is represented with a larger dot, and larger bars represent the averages of three means (*n* ≥ 30 for group). (K) The proposed model by which RAD51-mediated repair processes maintain genomic integrity. When MMS induces DNA double-strand breaks, high-expressed RAD51 efficiently responds to induce DNA replication and DNA repair. RAD51 effectively localizes to DNA break sites, where it interacts with RPA. EXO1 (exonuclease 1).

To investigate whether the highly expressed RAD51 enhances the HR-based repair of MMS-induced DSBs, we initially compared the EF1α and CMV promoters for efficient express RAD51 gene expression. While both promoters exhibited substantial strength in mammalian cells, the EF1α promoter proved more effective than the CMV promoter in driving RAD51 gene expression in HEK293T cells (Figure 3A). Consequently, the RAD51-EF1α plasmid was utilized to activate RAD51 in all experiments. Given that overexpressed RAD51 can induce the repair of DNA breaks, we examined the expression levels of γH2AX, phosphorylation of H2AX histones at the serine 139 residue, results in γH2AX formation, serving as indicator of DSB sites.^25^^,^^26^ Following MMS treatment, γH2AX expression levels were increased by 7.74-fold compared with empty vector transfected control (Ctrl) cells, while RAD51 overexpression resulted in a 2.07-fold reduction in MMS-stimulated γH2AX levels (Figures 3B and S3). DNA damage induced by MMS stimulate cell-cycle arrest in the S/G2 phase, providing time for DNA repair. Following MMS treatment, the S/G2 phase ratio increased by 1.40-fold in Ctrl cells and was further increased by 1.65-fold in RAD51-overexpressed cells. In the absence of MMS, the proportion of the S/G2 phase in Ctrl cells resembled that in RAD51 overexpressed cells (Figures 3C and 3D). Thus, abundant RAD51 under MMS-mediated DNA damage stress enhances DNA repair efficiency by activating the S/G2 checkpoint.

To prove whether RAD51 overexpression can directly reduce damaged DNA, a comet assay was performed using HEK293T cells (Figure 3E). The MMS-treated cells exhibited a notable increase in the number of cells exhibiting DNA tail moment. The DNA tail moments in Ctrl+MMS group were approximately 2.00-fold longer than those in the Ctrl group, while the RAD51+MMS group exhibited a 0.69-fold reduction compared to the Ctrl+MMS group (Figures 3E and 3F).

Cell viability was further compared between Ctrl+MMS and RAD51+MMS groups. The RAD51+MMS group demonstrated significantly lower percentages of apoptotic and dead cells (30.5%) compared to the Ctrl+MMS group (54.3%). Moreover, the percentage of live cells in the RAD51+MMS group (68.4%) was higher than that in Ctrl+MMS group (45.6%) (Figures 3G and 3H). Therefore, RAD51 overexpression promoted effective recovery from DNA fragmentation and apoptotic cell death induced by MMS-stimulated DNA damage.

Immunofluorescence (IF) staining for γH2AX was performed to estimate the presence of DSB sites under DNA damage stress. The number of γH2AX and RAD51 foci per nucleus was significantly higher in MMS-treated Ctrl cells than in untreated Ctrl cells. The increased γH2AX levels in MMS-treated Ctrl cells were significantly reduced by RAD51 overexpression (Figures 3I and 3J). Collectively, our results indicate that RAD51 promptly responds to and is recruited to DSB sites, promoting precise DNA repair and replication through an enhanced HR process (Figure 3K).

### Combination of RAD51 overexpression and SCR7 treatment improves HR efficiency

**Figure 4:**
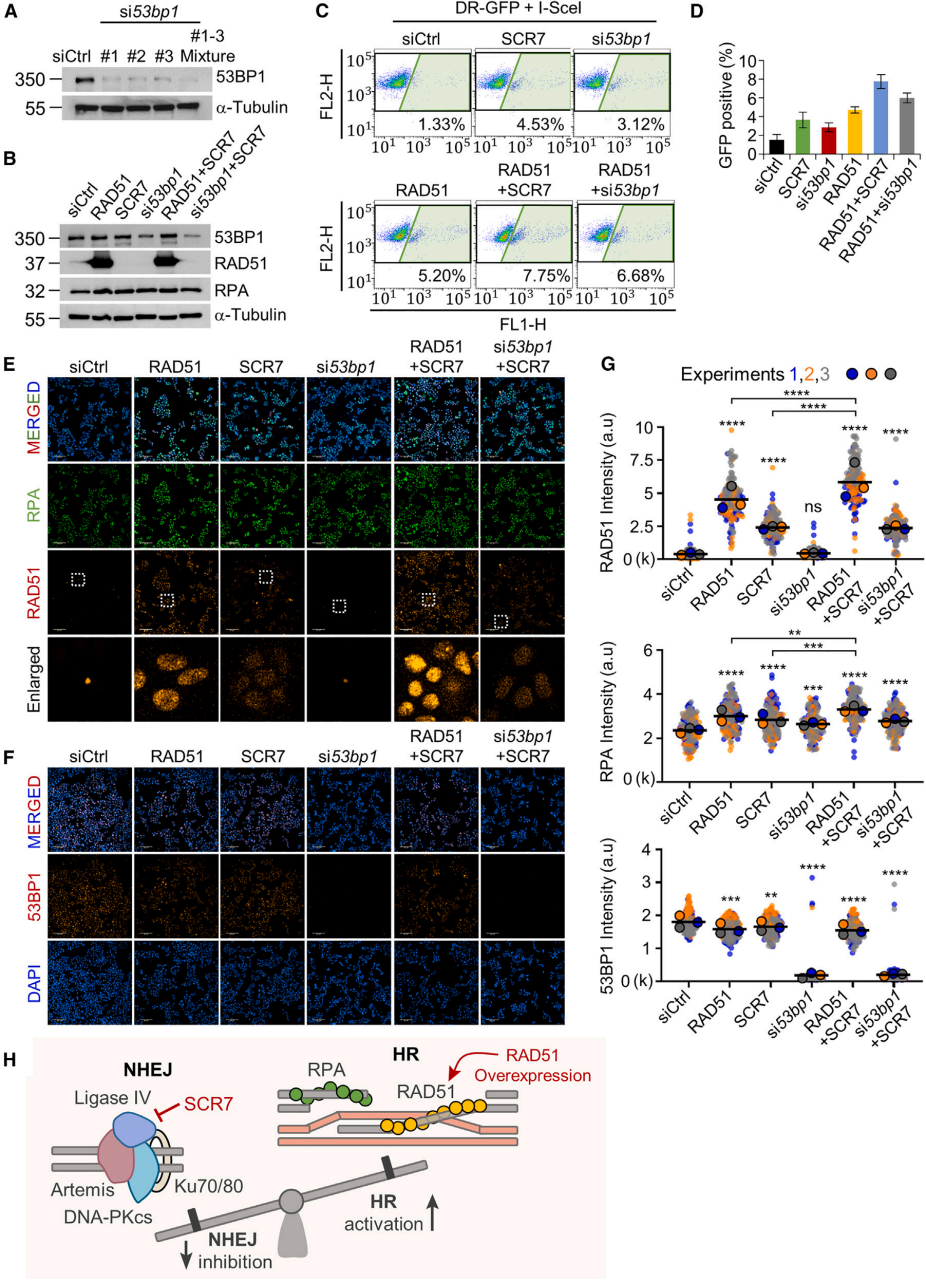
Regulation of HR pathway by RAD51 induction and NHEJ inhibition (A) Analysis of 53BP1 knockdown efficiency. 53BP1 expression in HEK293T cells was inhibited by siRNA pool against 53BP1 (si*53bp1*). A non-targeting siRNA (siCtrl) was used as a control gene. (B) Analysis of the expression pattern of HR/NHEJ-related proteins in response to various regulation factors of the HR/NHEJ pathway. (C) Analysis of HR repair assay. The effect of HR/NHEJ-related factors on HR pathway activation was assessed by GFP reporter-based quantification in pDR-GFP-stable HEK293T cells transfected with a pCBASceI plasmid. (D) The proportions of the GFP-positive HEK293T cells in (C) were quantified by FlowJo software. Error bars denote the mean ± SD. (E and F) Immunofluorescence analysis of HR-related proteins in HEK293T cells during the interphase stage. The nuclei were stained with anti-RPA, anti-RAD51, and anti-53BP1. Scale bars represent 100 μm. (G) Fluorescence intensity quantification of RAD51, RPA, and 53BP1 protein signals per nucleus in HEK293T cells. Values represent the mean with SD. ∗∗*p* < 0.01; ∗∗∗*p* < 0.001; ∗∗∗∗*p* < 0.0001; ns, not significant; paired two-tailed t test. (H) The proposed model by which RAD51 overexpression and inhibition of DNA ligase IV by SCR7 promote activation of the HR repair process.

Precise genome editing is achievable by activating the HR repair pathway using CRISPR-Cas9-induced DSBs.^5^ However, the efficiency of the HR pathway is limited by its competition with alternative DSB repair pathways, such as the NHEJ pathway.^27^ In this study, we used strategies to improve HR efficiency by introducing a RAD51 overexpression vector or incorporating 53BP1-specific siRNA and SCR7 to inhibit the NHEJ pathway. Initially, HEK293T cells were transfected with three different siRNAs targeting 53BP1, a pro-NHEJ factor impeding DNA end resection. The mixture of three siRNAs targeting different exons of 53BP1 was shown to be the most effective in inhibiting 53BP1 expression (91.25%) (Figures 4A and S4A). Cells treated with RAD51, SCR7, or si*53bp1* showed increased RPA expression levels, and the combination treatment with RAD51 and SCR7 showed the highest expression levels of RPA, suggesting the activation of the HR-mediated repair process (Figures 4B and S4B). The effects of those HR inducers and NHEJ inhibitors on HR repair activity were assessed using a DR-GFP reporter assay. In comparison to siCtrl cells, treatment with SCR7, si*53bp1*, or RAD51 overexpression alone elevated HR activity. Particularly, the combination treatment of SCR7 or si*53bp1* with RAD51 overexpression markedly increased HR activity. Cells treated with both RAD51 and SCR7 exhibited the highest HR activity (7.75%) compared to the other groups (Figures 4C and 4D). Additionally, we have examined the HR activity improved by RS-1, known as a RAD51 agonist.^28^ In response to 7.5 μM RS-1, HR efficiency increases by 3.41%, but in combination treatment, RS-1+SCR7 (5.97%) exhibits lower efficiency compared to RS-1+RAD51 (7.75%) and SCR7+RAD51 (9.16%). (Figure S4C). These results show that RAD51 overexpression combined with NHEJ inhibitors is the most effective approach to increase HR repair activity.

During the early steps of HR, the newly generated DSB ends were processed to yield long ssDNA overhangs, prompting engagement by RPA. Subsequently, RAD51 replaced RPA, participating in DNA strand exchange and homology strand search, playing an essential role in the HR process.^29^^,^^30^ Therefore, we investigated whether increased HR activity could enhance the levels of the ssDNA-binding protein RPA. As expected, the fluorescence intensity levels of RPA per nucleus were significantly higher in all groups compared to the siCtrl group, indicating activation of the HR repair pathway across all groups stimulated by the HR inducer and/or NHEJ inhibitors (Figures 4E and 4G). In addition, RAD51 intensity levels were significantly increased in SCR7-treated cells and SCR7+si*53bp1*-treated cells compared to those in siCtrl cells, with no discernible RAD51 expression in si*53bp1*-transfected cells. Notably, RAD51 levels in RAD51+ SCR7-treated cells were 1.3-fold higher than those in RAD51-treated cells (Figure 4G). Consequently, SCR7 treatment not only enhanced HR activity by increasing RAD51 expression levels but also by inhibiting the NHEJ pathway. The RPA-associated HR process exhibited an increase in HEK293T cells treated with the combination compared to those treated with either RAD51 overexpression or SCR7 alone (Figures 4E–4G).

Meanwhile, in contrast to RAD51 overexpression or SCR7 treatment, siRNA-mediated knockdown of 53BP1 reduced the cell growth rate by more than 50%; thus, 53BP1 siRNA does not appear to be suitable as an inhibitor of the NHEJ pathway (Figure S4D). These data demonstrate that SCR7 treatment is more effective than siRNA-mediated 53BP1 knockdown in activating the HR mechanism. In addition, IF analysis of 53BP1 showed that treatment with RAD51, SCR7, or RAD51+ SCR7 reduced the expression levels of 53BP1 protein, indicating that RAD51 overexpression may also contribute to the suppression of the NHEJ pathway (Figures 4F and 4G). Taken together, our results suggest that the combined treatment with RAD51 and SCR7 leads to increased RAD51 expression, ssDNA-binding activity, and decreased NHEJ, thereby resulting in improved HR-based DNA repair (Figure 4H).

### High HR efficiency achieved through the combined treatment with RAD51 and SCR7 significantly improves genome-editing efficiency

In a previous study,^18^ we developed an all-in-one Lenti-CRISPR-Cas9 vector system containing RAD51 coding region, a single gRNA cassette, an FLAG tag, and an EGFP reporter gene.^18^ To directly investigate the impact of RAD51 on CRISPR-Cas9-mediated genome editing, we established heterogeneous cells and stable HEK293T cell and hiPSC lines including gRNA targeting the human glyceraldehyde-3-phosphate dehydrogenase (hGAPDH) or human ataxia telangiectasia mutated (hATM), using the aforementioned vector system (Figure S5A).

**Figure 5:**
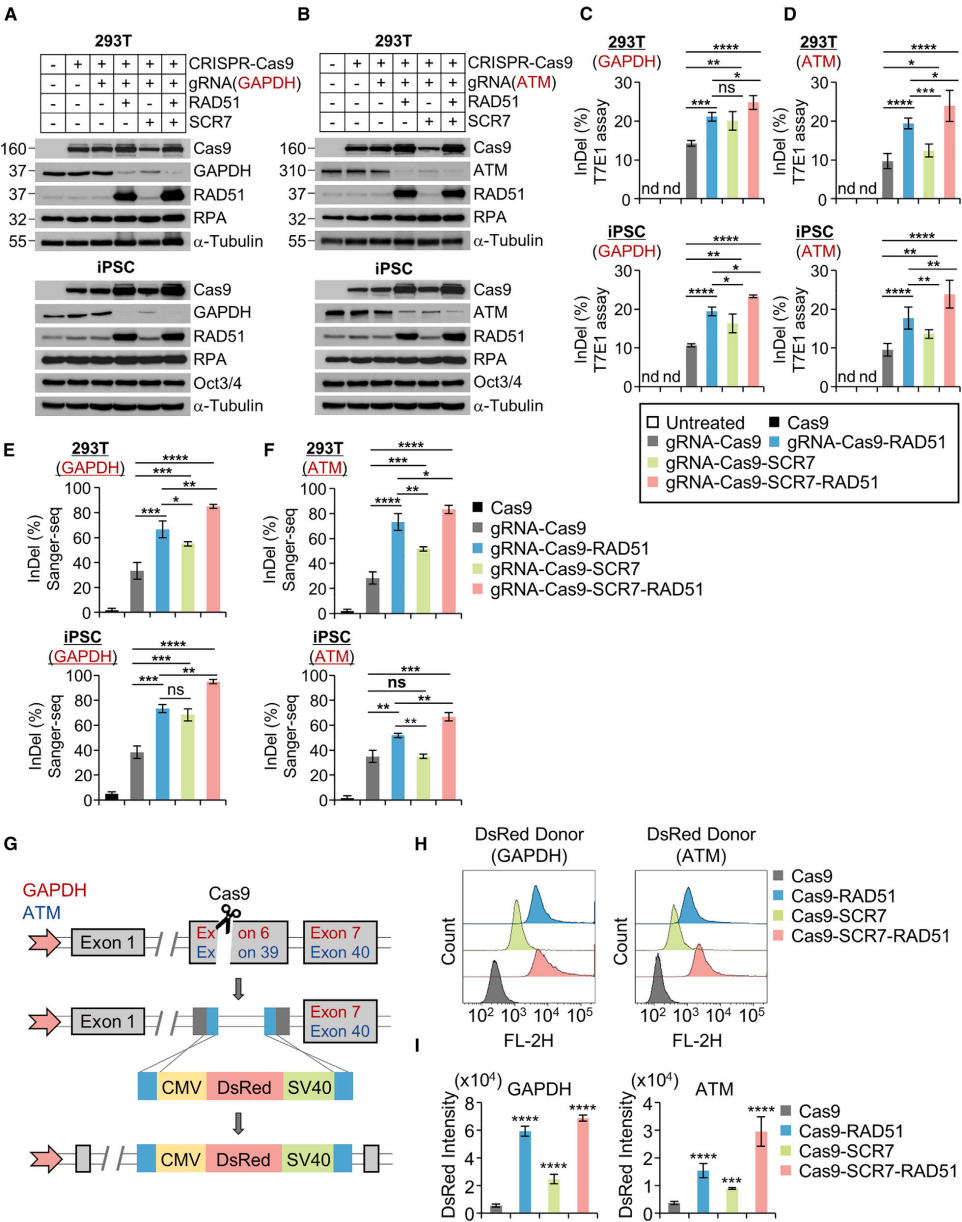
Relationship of genome modification using the CRISPR-Cas9 system with the activation of RAD51-induced HR process (A and B) Analysis of gene editing efficiency by CRISPR-Cas9 against GAPDH and ATM in HEK293T cells and hiPSCs stably expressing Cas9 or Cas9-RAD51. The expression level of GAPDH was quantified by immunoblotting assay, with α-tubulin and OCT3/4 used as a reference control for normalization and stemness marker, respectively. (C and D) T7E1 endonuclease assay for evaluating the InDel percentage. T7E1 analysis compared the generation of gene modification at the GAPDH and ATM locus by the CRISPR-Cas9 system in HEK293T cells and hiPSCs. The InDel percentage was measured as the ratio of the digested band to the undigested band (Figures S6A and S6B). (E and F) Analysis of genomic mutation by Sanger sequencing. The value was calculated by the proportion of modified sequences to the targeted-GAPDH and ATM locus (*n* = 20 for each condition. (G) The knockin system of dsRed cassette in HEK293T cells. Guide RNA targeting the terminal region of exon 39 (hATM gene) or exon 6 (hGAPDH gene) cuts that locus, promoting genomic integration of the dsRed though homologous recombination. (H) FACS analysis describes the cell number according to the amount of dsRed intensity in each condition (I) Quantitative analysis of dsRed fluorescence measured in Figure 5H. Error bars denote the mean ± SD (*n* = 3). ∗∗∗*p* < 0.001; ∗∗∗∗*p* < 0.0001; paired two-tailed test.

In heterogeneous cells, the inhibition of GAPDH gene expression by CRISPR-Cas9 is shown to be 9.9% for gRNA-Cas9, 36.1% for Cas9-RAD51, 29.6% for Cas9-SCR7, and 37.0% for Cas9-RAD51-SCR7, compared to the Ctrl (Figures S5B and S5C). To accurately assess the enhancement of CRISPR-Cas9 efficiency by HR activators or NHEJ inhibitors, we analyzed the knockout level of GAPDH or ATM in HEK293T cells and hiPSCs stably expressing all-in-one Lenti-CRISPR-Cas9 (Figure S5A). Immunoblot analysis revealed that the expression levels of the GAPDH protein were slightly reduced in Cas9-gRNA-expressing stable cells compared to those in Ctrl cells (untreated cells), but the reduction was not significant. However, significant gene suppression efficiency was observed in HEK293T cells and hiPSCs under RAD51 alone (77.6%, 93.6%) or SCR7 alone (68.5%, 83.3%), with RAD51+SCR7 (87.5%, 94.8%) showing that combination treatment led to the highest GAPDH suppression efficiency (Figures 5A and S5D). In HEK293T cells and hiPSCs introduced with gRNA targeting ATM, ATM expression decreases by 26.4% for HEK293T cells and 46.4% for hiPSCs (Figures 5B and S5E). However, under RAD51, SCR7, and RAD51+SCR7, ATM expression inhibition increases by over 83%. Similar to the GAPDH suppression level, the combination treatment of RAD51+SCR7 exhibits the highest CRISPR-Cas9 efficiency in the ATM targeting site (Figures 5B and S5E).

Subsequently, we explored the percentage of indels induced by CRISPR-Cas9 with gRNA targeting GAPDH and ATM using the T7 endonuclease 1(T7E1) mismatch cleavage assay, a method employed to detect InDel mutations at the genomic target site and estimate genome-editing efficiency. In both cell types, the T7E1 analysis for GAPDH demonstrated elevated InDel percentages in Cas9-gRNA-RAD51 (6.93% for HEK293T, 8.76% for hiPSC), Cas9-gRNA+SCR7 (5.88% for HEK293T, 5.65% for hiPSC), and Cas9-gRNA-RAD51+SCR7 cells (10.62% for HEK293T, 12.64% for hiPSC) compared to that Cas9-gRNA cells. Conversely, groups without gRNA did not exhibit the digested band (Figures 5C and S6A). Moreover, the T7E1 analysis for ATM showed improved InDel percentage in Cas9-gRNA-RAD51 (9.77% for HEK293T, 8.12% for hiPSC), Cas9-gRNA+SCR7 (2.77% for HEK293T, 4.04% for hiPSC), and Cas9-gRNA-RAD51+SCR7 cells (14.28% for HEK293T, 14.92% for hiPSC) compared to that Cas9-gRNA cells (Figures 5D and S6B). We further performed Sanger sequencing to validate whether RAD51 improve genome-editing efficiency. Aligned sequences represented the gene knockdown and InDel mutations that occurred by each plasmid expression in HEK293Ts and hiPSCs, respectively (Figures S7A–S7D). The genome-editing efficiency, measured by the fraction of wild-type GAPDH sequences to total sequences, was increased in Cas9-gRNA-RAD51 (2.0-fold for HEK293T, 1.78-fold for hiPSC), in Cas9-gRNA+SCR7 (1.66-fold for HEK293T, 1.65-fold for hiPSC), and in Cas9-gRNA-RAD51-SCR7 (2.57-fold for HEK293T, 2.5-fold for hiPSC) compared to Cas9-gRNA (Figure 5E). In wild-type ATM targeting sites, analysis of InDel formation by Sanger sequencing showed increased proportion of InDel mutation in Cas9-gRNA-RAD51 (2.59-fold for HEK293T, 1.47-fold for hiPSC), in Cas9-gRNA-SCR7 (1.82-fold for HEK293T, 1.03-fold for hiPSC), and in Cas9-gRNA-RAD51-SCR7 (2.94-fold for HEK293T, 1.91-fold for hiPSC) compared to Cas9-gRNA (Figure 5F). These findings indicate that combined RAD51-SCR7 stimulation not only accelerated CRISPR-Cas9-mediated precise genome editing but also enhanced the efficiency of CRISPR-Cas9-mediated target gene disruption.

### RAD51 and SCR7 increased the efficiency of exogenous gene insertion

To analysis the knockin regulation efficiency of RAD51, SCR7, and combination treatment, we designed donor templates for the multiple endogenous regions in HEK293T cells. We designed the gRNA that can bind directly to terminal sequences of the hATM gene exon 39 and hGAPDH gene exon 6.^18^^,^^31^ The donor template contains the fluorescent marker gene (dsRed) following the CMV promoter flanked by about 680-bp homology arms for ATM and 800-bp homology arms for GAPDH (Figure 5G). Under each condition, the knockin efficiency was measured by analyzing the level of dsRed fluorescent signals. Flow cytometry analysis showed that the distribution of the histogram peaks depends on the dsRed fluorescent intensity (Figure 5H). RAD51, SCR7, and their combination treatments improve the knockin efficiency at the endogenous GAPDH sites by 10.7-fold, 3.23-fold, and 12.48-fold, respectively, compared to Cas9-gRNA. Similarly, at the endogenous ATM sites, knockin efficiency was increased by 4.26-fold, 2.54-fold, and 8.28-fold, respectively (Figures 5I and S8A–S8D). Thus, these results demonstrated that combination treatment of RAD51 and SCR7 effectively can improve gene integration using a knockin process in multiple genomic loci.

### Elevated expression of exogenous RAD51 and SCR7 prevents accumulation of R-loops

**Figure 6:**
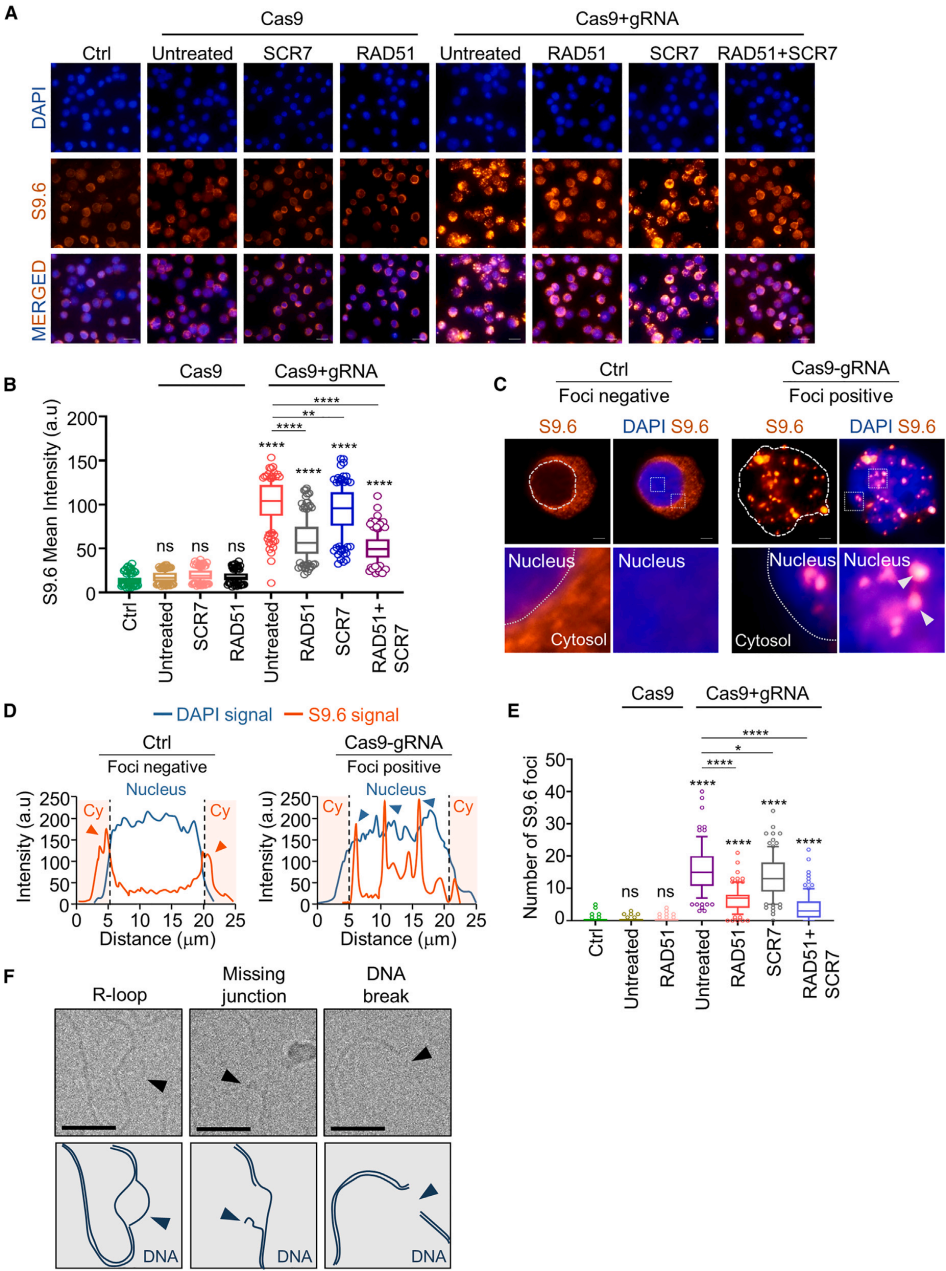
Change in R-loop formations induced by HR regulation (A) Immunofluorescence analysis of R-loop formation using S9.6 antibody during the interphase stage in stable HEK293T cells containing Cas9. Endogenous R-loop intensity was evaluated by fluorescence microscopy, with the scale bar set at 10 μm. (B) Mean intensity of S9.6 per nucleus. The box and whisker graph indicates the mean (single line in the boxplot), quartiles (box, 25%–75% value), whiskers (lines extending from the box, 10%–90%), and outliers (circle, values beyond the boxplot range) from three independent biological replicates (*n*≥ 100 for each condition). a.u., arbitrary unit. (C) R-loop focus formation detected by anti-S9.6. Scale bars represent 2.5 μm. (D) Intensity profiling in the nucleus. Images were analyzed using NIS advanced software (Nikon). a.u., arbitrary unit. (E) Numbers of R-loop focus formation and the ratio in the nucleus. Focus count was performed using NIS advanced software (Nikon) and GraphPad Prism (version 9.0). ∗*p* < 0.05; ∗∗∗∗*p* < 0.0001; ns, not significant; paired two-tailed t test. (F) Transmission electron microscopy analysis of R-loops on genomic DNA extracted from HEK293T cells with *in vitro* RNase H digestion. Representative electron microscope images of R-loops (indicated by the black arrows) found on gDNA from HEK293T cells. Scale bars represent 25 μm.

In the context of the CRISPR-Cas9 system, R-loop generation is integral to genome editing; however, their resolution is imperative for DNA repair, as persistent R-loops can result in DNA damage, genome instability, and reduced editing efficiency.^32^^,^^33^ Based on these results, we tested whether RAD51 and SCR7 regulate the stabilization and resolution of R-loop formation in HEK293T cells. To analyze this, we used the S9.6 antibody to identify RNA-DNA complexes and other RNA structures. As shown in Figure 6A, highly induced RNA-DNA hybrids were identified by S9.6 in Cas9-gRNA-expressed cells. The increased S9.6 intensity in Cas9-gRNA cells was significantly reduced by 1.7-fold with RAD51 overexpression and 2.0-fold with combined RAD51 and SCR7 treatment, suggesting that RAD51 or the combination of RAD51 and SCR7 could promote R-loop resolution during the Cas9-generated DSB repair process (Figures 6A and 6B). Moreover, we analyzed the number of S9.6-labeled R-loop foci formed in the nuclei of single cells. Cas9-gRNA cells displayed a marked increase in R-loop foci formation in the nucleus, whereas Ctrl cells showed no foci formation in the nucleus (Figures 6C and 6D). The number of R-loop foci found in the nuclei of Cas9-gRNA cells significantly decreased by 2.3-fold in Cas9-gRNA-RAD51 cells and by 3.8-fold in Cas9-gRNA-RAD51+SCR7 cells. SCR7 treatment had minimal effect on R-loop resolution (Figure 6E). These findings indicate that RAD51 alone or in combination with RAD51 and SCR7 prevents the accumulation of Cas9-gRNA-mediated R-loop and promotes R-loop resolution, facilitating DSB repair by HR. Consequently, RAD51 and SCR7 treatment exhibited synergistic effects in Cas9-gRNA-expressed HEK293T cells, contributing to the maintenance of genome stability and the enhancement of HR-mediated precise gene editing.

## Discussion

CRISPR-Cas9 constitutes a potent genome-editing system where guide RNA facilitates the recruitment of Cas9 complexes to a target site in the genome. The nuclease activity of these complexes induces DSBs, subsequently repaired through employing two main DNA repair processes.^1^^,^^34^ The choice of DNA repair pathway depends on the cell-cycle stage, with HR proving efficient in the S/G2 phase and NHEJ predominating during the G1 phase.^35^ Accurate genome editing by the CRISPR-Cas9 system is based on the HR process; however, genome editing of several cells has proven challenging as they may undergo apoptosis and/or predominantly utilize NHEJ for DNA damage repair.^35^^,^^36^ Moreover, the rate of repair by HR is typically slower compared to the more rapid but less precise NHEJ-mediated repair.^9^ Recently, promising methods have been developed to enhance the efficiency of HR machinery-dependent genome editing in several cell types. These methods involve delivering NHEJ inhibitors, boosting the HR process, and improving the concentration of the repair DNA template.^8^^,^^10^^,^^37^ Nevertheless, there is currently no mechanistic explanation for why enhancing the recombination-based pathway enables precise genome editing.

In this study, we implemented a strategy to enhance the efficiency of recombination-based mechanisms by elevating RAD51 expression through the all-in-one CRISPR-Cas9-RAD51 system and/or by inhibiting the NHEJ pathway. We investigated whether HR-dependent RAD51 contributes to repairing DNA breaks upon RAD51 expression inhibition. Total RNA-seq analysis of global transcript levels showed that RAD51 loss impeded cell-cycle progression and led to low HR activity by inhibiting the G2/M transition in HEK293T cells. Inefficient HR machinery affects the post-replication step via the ssDNA gap repair process, consequently disrupting complete DNA replication and potentially inducing cell death. Therefore, dysregulation of the DNA repair system may compromise the efficiency of HR-mediated CRISPR-Cas9 genome editing.

**Figure 7:**
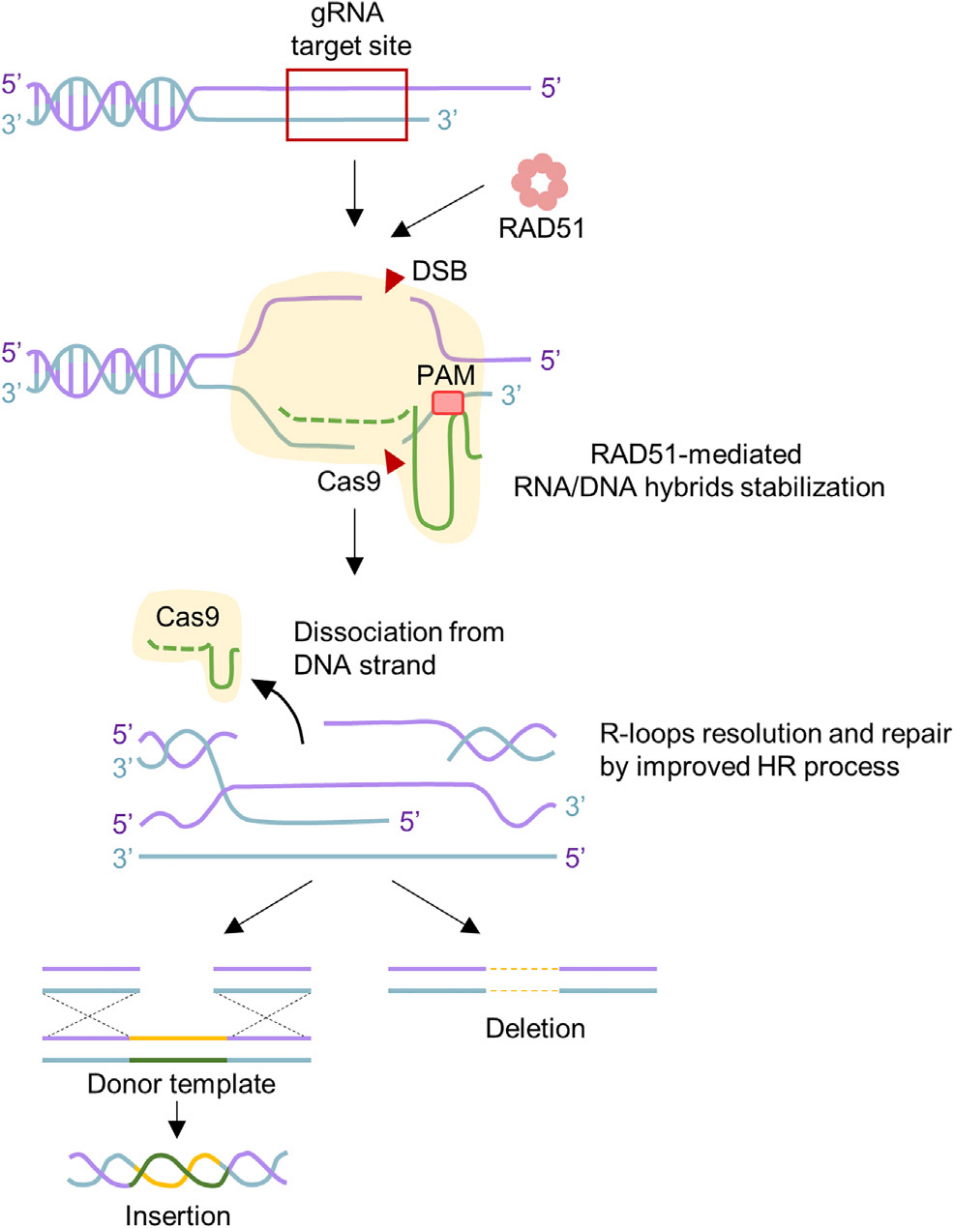
A proposed model for enhancing the CRISPR-Cas9 system by overexpressed RAD51 Cas9-gRNA complex binds to the DNA strand in PAM-dependent manner. gRNA invades double-strand DNA and forms the RNA/DNA hybrid with the target strand while dislocating the opposite single-strand DNA. Elevated expression of exogenous RAD51 improves DSB repair through efficient HR pathway. It leads to the dissociation of the Cas9 complex from DNA strand and the resolution of the Cas9-mediated R-loop, ultimately enhancing genome-editing efficiency.

The CRISPR-Cas9-RAD51 system, an HR-based genome-editing strategy, showed that the elevated expression of exogenous RAD51 increased the proportion of the HR pathway during DNA damage repair and replication. In addition, this pathway is more frequently utilized when the NHEJ pathway is inhibited by SCR7 under RAD51 overexpression conditions. Interestingly, we found that improved HR activity induced by RAD51, SCR7, and their combination at multiple genome loci not only enhances knockin efficiency but leads to higher gene knockdown effects compared to Cas-gRNA alone. We postulate two hypotheses based on the results of enhanced HR activity by RAD51-SCR7 and the promoted efficiency of CRISPR-Cas9-based gene knockin and knockdown: (1) maintenance of genomic integrity via efficient DNA repair and (2) high stabilization of RNA/DNA hybrid and R-loop resolution through improved HR activity (Figure 7).

R-loops are three-stranded structures consisting of RNA/DNA hybrids and ssDNA displaced from a non-template strand.^38^ Thus, R-loops are thermodynamically sensitive and unstable. Upon formation of R-loops, the Cas9-gRNA complex interacts with them, occurring in target DNA sequences that bear gRNA segment complementarity.^39^ Conformational changes in the Cas9 structure lead to the construction of a complete R-loop and the formation of a DNA recognition-competent structure, activating the Cas9 RuvC and HNH nuclease domains for the cleavage of the target strand and non-target DNA strands (NTSs).^40^^,^^41^ Thus, successful genome editing by CRISPR-Cas9 at the target DNA strand necessitates stable establishment and dissolution of the Cas9-R-loop structure.

DNA/RNA hybrids form through the Cas9 gRNA complex, and ssDNA dissociates from the original DNA strand. We found that under exogenous expression of RAD51, Cas9 complex may potentially interact with RAD51 (Figure S9), but more specific clues are needed on how RAD51 can bind to Cas9 subunits to understand the functional significance of the direct RAD51-Cas9 complex interaction. Nevertheless, these observations imply that high RAD51 levels may form nucleoprotein filaments with ssDNA of the displaced NTS and enable the stabilization of Cas9-regulated R-loops by inhibiting dsDNA reannealing. In addition, elevated RAD51 expression effectively resolved aberrant R-loop accumulation in CRISPR-Cas9 genome system (Figure 6E). R-loops play an essential role in transcription, replication, and DNA damage repair, but if these structures cannot be resolved through the replication stress responses of DNA repair and R-loop homeostasis, they cause inhibition of DSB resection and genome rearrangements.^33^^,^^42^ This establishes a positive feedback model where insufficient DSB resection leads to additional R-loop accumulation.^38^ Buildup of R-loops during cellular stress responses unveils potential mechanisms contributing to efficient genome editing. Thus, it can be problematic to the CRISPR-Cas9 system as blocks to efficient replication and transcription that cause the DNA damage response. Here, we report a significant reduction in the ratio of R-loops formed by the Cas9 complex, with Cas9-gRNA-RAD51 (58.0%) and Cas9-gRNA-RAD51-SCR7 (74.1%) compared to Cas9-gRNA alone (Figures 6C–6F). These data suggest that increased HR activity by RAD51 and SCR7 facilitates efficient DNA replication and induces stable resolution of R-loops generated during the genome-editing process (Figure 7). RAD51 polymerizes to ssDNA to form nucleoprotein filaments, which are crucial intermediates in HR resection. These RAD51 helical filaments are advantageous for the initiation of invasion and homology searching for target loci. Because RAD51 is important in this step, increased RAD51 binding early after DSB formation may be involved in efficient R-loop removal by promoting the recombination-based repair process. Taken together, both HR- and NHEJ-regulated genome editing can be promoted by CRISPR-Cas9-gRNA’s effective enhancement of RAD51-mediated DSB repair and R-loop removal.

This study focused on strategies to enhance CRISPR-Cas9 editing efficiency by regulating HR/NHEJ activity under RAD51 and/or SCR7 treatment. RAD51 overexpression enhances HR efficiency, and SCR7 inhibits NHEJ by inhibiting the function of DNA ligase IV, thereby enhancing HR efficiency. However, DNA ligase IV dysfunction by SCR7 can provide a cause of disturbance in the immune system and genetic diseases such as growth failure, pancytopenia, hypogonadism, and immunodeficiency. Notably, RAD51 overexpression can cause abnormal expression of genes associated with cancer development. Therefore, methods to enhance the efficiency of gene modification by regulating HR/NHEJ may be a potential limitation for future gene and cell therapy studies. In *in vitro* or *in vivo*, controlling the level of RAD51 expression can be quite challenging, and achieving sustained protein expression can be extremely difficult. As an alternative, we found that treating with SCR7 or RS-1 can enhance gene-editing efficiency, and combining these with other small molecules can induce more precise gene editing. The use of small molecules *in vivo* is advantageous over protein overexpression systems because it allows for the regulation of timing and optimal concentration required for gene editing. Enhancing gene editing efficiency through small-molecule co-treatment will be an important strategy for future *in vivo* and gene therapy research.

We confirmed that RAD51 and Cas9 complexes physically interact through immunoprecipitation analysis. Based on these results, we will investigate the functional relationship between RAD51- and Cas9-specific domains and proceed with a functional study of genome-editing efficiency through the regulation of protein interaction sites. In addition, it will be interesting to test whether RAD51 can recruit BRCA1/2 and RPA with Cas9 complex to DSB sites and enhance genome-editing efficiency by the CRISPR-Cas9. Subsequent studies will suggest new research directions for CRISPR-Cas9 studies through the discovery and function identification of proteins that can regulate Cas9 in eukaryotic cell models.

## Materials and methods

### Cell lines

HEK293T cells, primary human dermal fibroblasts (HDFa), human colon cancer HCT116 cells, LUHMES cells, and hiPSCs were purchased from ATCC. HEK293T cells and HDFa were cultured in Dulbecco’s modified Eagle’s medium (DMEM; Hyclone, SH30243.01) supplemented with 10% fetal bovine serum (FBS; Hyclone, SH30919.03) and 1% penicillin/streptomycin (P/S; Hyclone, SH40003.01). HCT116 cells were cultured in DMEM/low glucose (Gibco, 11885-084) supplemented with 10% FBS and 1% P/S. LUHMES cells were grown on plates precoated with 50 μg/mL poly-L-ornithine (Sigma; Cat. P3655) and 1 μg/mL human plasma fibronectin (Sigma, F0895). Cells were cultured in DMEM/F12 (Gibco, 30–2006) supplemented with 1% N2 (Gibco, 17502-048) and 40 ng/mL human recombinant basic FGF (Gibco, 13256-029). hiPSCs were maintained on a feeder-free condition in mTeSR1 medium (Stemcell, #85850). The cells dissociated into single cells with Accutase (Stemcell, #07920) at 37°C for 5 min and seeded onto a Matrigel-coated culture dish at 50,000 cells/cm^2^ in mTeSR1 supplemented with 10 mM Y27632 (Stemcell, #72302). Cells were then cultured in mTeSR1 without Y27632 for 2 days. All cells were cultured at 37°C in a humidified incubator with 5% CO_2_.

### CRISPR-Cas9 gRNA and donor vector design

Design of the gRNA and plasmid construction was prepared as described previously.^18^ Complementary oligonucleotides of hGAPDH (5′-CACCTTCCTCACCTGATGATCTTGG-3′) and hATM (5′-CACCGACTACATGAGAAGACAAAAG-3′) were ligated into lentiCRISPRv2 vector (Addgene, #52961) that was digested with restriction enzyme BsmBl (NEB, R0739S). Subsequently, either EGFP without hRAD51 or EGFP with hRAD51 was cloned into the lentiCRISPRv2 vector containing a puromycin resistance cassette. Four plasmids were used in these experiments: (1) lentiCRISPRv2-Cas9-EGFP, (2) lentiCRISPRv2-Cas9-EGFP-RAD51, (3) lentiCRISPRv2-Cas9-gRNA-EGFP, and (4) lentiCRISPRv2-Cas9-gRNA-EGFP-RAD51. The genetic construct for DNA knockin was a vector harboring the dsRed cassette flanked with homology arms. A DNA fragment of 1.6 kb from the hGAPDH gene, and 1.4 kb from the hATM gene, encompassing the target locus, was amplified and inserted into the plasmid backbone. Inverse PCR was employed to introduce restriction enzyme sites proximal to the target locus, facilitating the insertion of the dsRed cassette. The resulting dsDNA template comprised the dsRed cassette flanked by homology arms, obtained through PCR amplification.

### Stable cell lines

To establish a stable cell line expressing Cas9 or Cas9-RAD51, 3 μg all-in-one LentiCRISPRv2 plasmid was transfected with 20 μg polyethylenimine (PEI; Sigma, 408727) into the HEK293T cells and hiPSCs. Cells were incubated for 24 h, and the media were replaced with fresh media 24 h post transfection. Subsequently, 1 μg/mL puromycin (Sigma, P8833) was added for selection for 2 weeks, and then GFP-positive cells were sorted by a BD FACS Aria lll (Becton Dickinson), using non-transfected cells to define the background fluorescence level. The sorted cells were harvested and used in these experiments.

### Fluorescence-activated cell sorting

To analyze the CRISPR-Cas9 knockin efficiency, HEK293T cells and hiPSCs were harvested and washed with ice-cold phosphate-buffered saline (PBS). Cells were fixed with 70% ethanol at −20°C for overnight. Fixed cells were analyzed using Gallios flow cytometer (Beckman Coulter) and quantified with the Kaluza C analysis software.

### Antibodies

The following primary antibodies were used in this article: anti-GAPDH (Abcam, ab8245; 1:10,000), anti-α-tubulin (Abcam, ab4074; 1:10,000), anti-γH2AX (CST, #2577; 1:1,000), anti-FLAG (Merck Millipore, F1804; 1:5,000), anti-CRISPR-Cas9 (Abcam, ab191468; 1:5,000), anti-RPA (CST, #2208; 1:5,000), anti-RAD51 (Merck Millipore, PC130; 1:5,000), anti-53BP1 (Abcam, ab181616; 1:3,000), anti-RAD54 (Santa Cruz, sc-374598; 1:1,000), anti-ATM (Abcam, ab32420; 1:2,000), and anti-OCT3/4 (CST, #2840; 1:5,000). The following secondary antibodies were then used: HRP-AffiniPure goat anti-mouse IgG (Jackson IR, 115-035-003; 1:20,000), HRP-AffiniPure goat anti-rabbit IgG (Jackson IR, 111-035-003; 1:20,000), HRP-AffiniPure goat anti-rat IgG (Jackson IR, 112-005-003; 1:20,000), anti-rabbit TRITC (Jackson IR, 111-025-003; 1:250), anti-mouse TRITC (Jacson IR, 115-025-003; 1:250), anti-rabbit FITC (Jackson IR, 111-095-003; 1:250), and anti-rat FITC (Jackson IR, 112-095-003; 1:250).

### Immunoprecipitation and immunoblot

Samples were prepared as described previously.^43^ For immunoprecipitation assay, the cells were seed to a concentration of 1× 10^7^ cells/ml per 100-mm culture dish. The cells were washed by ice-cold 1× PBS to remove the extra serum and treated 0.3% Trypsin-EDTA for 1 min at 37°C. Subsequently, the cells were resuspended with ice-cold medium and centrifuged for 3 min. Cell pellets were resuspended with lysis buffer (25 mM Tris-HCl [pH 7.6], 150 mM NaCl, 0.1% NP-40, 0.1 mM EDTA, and 1% sodium deoxycholate) for 5 min on ice. After centrifugation (15,000 rpm for 5 min), the protein lysates were transferred to 1.5-mL tube and concentrated by the BCA protein assay. Soluble proteins (1 mg) were reacted with 1.5 μg of primary antibodies overnight and mixed with 25 μL 50% protein-A/G beads for 2 h at 4°C. The samples were centrifuged at 4,000 rpm for 1 min at 4°C, and the supernatants were removed. Protein-A/G beads were washed three times by washing buffer (10 mM HEPES, 2 mM MgCl2, 100 mM KCl, 1 mM DTT, 0.1% NP-40 in 1× PBS). After centrifugation (4,000 rpm, 1 min), A/G agarose beads were mixed with 2× SDS loading buffer and boiled for 3 min at 95°C heat block. The samples were analyzed by SDS-PAGE.

### Cell-cycle synchronization

For analysis of RAD51 expression pattern in HEK239T cells, cells were synchronized at G0-G1 phase by reacting with 2 μM thymidine (Sigma, 89270) for 24 h, after which the thymidine was washed and replaced a fresh medium. Afterward, the cells were reacted with a 2^nd^ thymidine and incubated for 24 h. The samples were washed with 1× cold PBS and cultured in fresh medium to release the cell-cycle progression from the 2^nd^ thymidine block. To obtain synchronization, duplicated cultures were collected after thymidine block release at 2.5-h intervals.

### Cell viability assay

Cytotoxicity of the MMS to the HEK293T cells was tested by using the cell counting kit-8 (CCK-8). Briefly, 1.5 × 10^4^ cells per well were seed in the 96-well plates and incubated with 5% CO_2_ at 37°C for 48 h. CCK-8 solution of 10 μL per well was added, followed by an additional incubation of 2 h and then analysis using the Multi-microplate reader (Biotech). The absorbance was measured at 450 nm. To analyze death, apoptosis, and live cells, HEK293T cells were briefly washed with cold PBS. Harvested cells were suspended in PBS containing 80 nmol/L thiazole orange (TO; Becton Dickinson, #349483) and 4.0 μmol/L PI (Becton Dickinson, #349483), and they were incubated for 10 min at room temperature. Cells were analyzed using an FACS Aria III flow cytometer (Becton Dickinson) and quantified with FlowJo software (FlowJo, LLC).

### Immunostaining

Cells were attached on poly-L-lysine-coated (Santa Cruz, sc-286689) round cover slips (Deckglaser, #0111520) and then fixed by 4% paraformaldehyde for 5 min. For membrane permeabilization, cells were treated with 0.1% Triton X-100 for 10 min and washed with PBS-T (0.1% Tween 20 in PBS solution) for 1 min three times. Cells were blocked with 5% BSA in PBS-T for 30 min and incubated for 1.5 h with primary antibodies (diluted in 2% BSA in PBS-T): anti-RAD51 (Merck Millipore, PC130; 1:500), anti-RPA (CST, #2208; 1:500), anti-γH2AX (CST, #2577; 1:200), anti-53BP1 (Abcam, ab21083; 1:500), and anti-S9.6 (Kerafast, ENH001; 1:200). Cells were washed with PBS-T three times and incubated for 1 h with the fluorescence-conjugated secondary antibodies (diluted in 2% BSA in PBS-T): anti-FITC (Jackson lab, 112-095-003; 1:500) and anti-TRITC (Jackson lab, 109-025-003; 1:500). Images were captured using the Eclipse Ti2 inverted microscope system (Nikon).

### HR DNA repair assay

HEK293T cells were transfected with the integrated HR reporter plasmid pDR-GFP (Addgene, #26475) and selected by puromycin of 1 μg/mL for 14 days to obtain pDR-GFP-positive cells. Subsequently, HEK293T cells stably expressing pDR-GFP were transfected with the endonuclease I-SceI producing plasmid pCBASceI (Addgene, #26477). After 48 h, cells were collected and fixed in 70% ethanol. The percentage of GFP-positive cells was analyzed by BD FACS Aria lll flow cytometry using FlowJo software.

### Small molecules

SCR7 pyrazine (MedChemExpress, HY-12742) was used in HEK293T cells and hiPSCs to inhibit NHEJ efficiency while simultaneously enhancing HR efficiency. Both HEK293T cells and hiPSCs were treated with 20 μM SCR7 for 48 h. RS-1 (RAD51-stimulatory compound 1; Sigma, R9782) was used as stimulator of the HR-mediated process. 7.5 μM RS-1 was treated in the cells for 48 h. The cells were washed by 1× PBS to remove the extra compounds and harvested.

### Comet assay

A neutral comet assay was performed.^22^ In brief, HEK293T cells were mixed with 1% low-melting temperature agarose (LMA) gel (Takara, 50101). The LMA gel was pipetted onto the slide glass. Once the LMA gel had solidified, the cells were immersed in cell lysis buffer (0.5 M Na2EDTA, 2% sodium lauroyl sarcosinate, 0.5 mg/mL Proteinase K [pH 8.0]) for 24 h at 37°C. The slides were submerged in 1× rinse buffer for 30 min at room temperature. Electrophoresis was conducted at 25 V, 8 mA, for 25 min. The slides were washed in an electrophoresis buffer (90 mM boric acid, 90 mM Tris, 2 mM Na2EDTA [pH 8.5]) for 3 min for three times. The slides were stained with 2.5 mg/mL propidium iodide in 1× PBS for 5 min and observed with a confocal microscope. The DNA tail moment was quantified using CASP software (1.2.3 beta2).

### RNA interference

A commercially available predesigned siRNA AccuTarget (Bioneer) specific to the target genes was used to knock down their endogenous expression in HEK293T cells. The siRNA pool included the following sequences: si*rad51*: 5′-GGUAAUCACCAACCAGGUA(dTdT)-3′, (1) si*53bp1*: 5′-CUGUGAAAGUUCUAGUGAA(dTdT)-3′, (2) si*53bp1*: 5′- GUGUCAUUACAGAUGUGUA (dTdT)-3′, and (3) si*53bp1*: 5′- GACUCUCCAGAAAUUCCUU (dTdT)-3′. The siRNAs were transfected with RNAiMAX Lipofectamine (Thermo, 13778150) according to the manufacturer’s protocol. The cells transfected with siRNAs were incubated in a serum-free medium for 24 h.

### Genomic DNA extraction and T7E1 mutation detection assay

Genomic DNA was isolated from cloned HEK293T and hiPSC lines using the Wizard Genomic DNA Purification Kit (Promega, A1120) according to manufacturer’s instructions. A region of the hGAPDH and hATM containing the target locus was amplified following a primer set: F-5′-ATCCCTCCAAAATCAAGTGGG-3′; R-5′-TGTGGTCTGCAAAAGGAGTGA-3′ for hGAPDH and F-5-GGCCTCAAGTGATCCGTC-3′; R-5′- GCCACAGATTTTGAGACCACT-3′ for hATM. The amplified PCR products were denatured at 95°C for 10 min and annealed through ramp down to 16°C at a cooling rate of 0.2°C/s in a Thermal cycler. T7 endonuclease 1 (T7E1; NEB, E3321) was then added to the annealed PCR product and incubated at 37°C for 15 min. T7E1 digestion products were electrophoresed on a 1.5% agarose gel. Gel images and quantification were analyzed using the GelDoc XR+ system (Bio-Rad, 1708195) and image lab software. The estimated gene modification was calculated with the formula {1 − [1 − (b + c)/(a + b + c)]^1/2^} × 100% (a, undigested band intensity; b and c, digested band intensity).^44^^,^^45^

### PCR product cloning and Sanger sequencing

Sanger sequencing was prepared as previously described.^18^ Briefly, purified PCR products from CRISPR-Cas9 vector expressing stable HEK293T cells and hiPSCs were cloned into vector using All in one TM PCR Cloning Kit (BIOFACT) according to manufacturer’s instructions. Sequencing reactions were analyzed on SnapGene.

### DNA preparation for Cryo-EM imaging

10 μg of this genomic DNA from HEK293T cells was digested with 50 U EcoRI-HF (NEB, R3101S) and 50 U PvuII-HF (NEB, R3151S) in NEB CutSmart buffer at 37°C for 4 h. To remove RNA moieties, the products were then digested with 0.25 μg/mL RNase A (Thermo Scientific, EN0531), 25 U/ml RNase T1 (Thermo Scientific, EN0541), 25 U/ml RNaseIII (NEB, M0245S) ± RNase H (NEB, M0297S) in NEB RNase H buffer, supplemented with 0.5 mM NaCl at 37°C for 2.5 h. The DNA was subsequently purified using the Slica Bead DNA Gel extraction kit (Thermo Scientific, K0513), according to the manufacturer’s protocol.

### Vitrified sample preparation for Cryo-EM

3 μL of the sample was applied to glow-discharged Quantifoil R1.2/1.3 Cu 300 grids (Quantifoil) and was flash frozen in liquid ethane using Vitrobot mark IV (Thermo Scientific) set at 100% humidity and 8°C for the preparation chamber and 3∼4 s blot time. Cryo-EM micrographs were imaged on Glacios microcscope (Thermo Scientific) operated at an accelerating voltage of 200 kV with a 70-μm C2 aperture. A Falcon IV direct electron detector was used to acquire images of the samples with a 100-μm objective aperture.

### RNA-seq library preparation and sequencing

Total RNA was isolated using the RNeasy mini kit (Qiagen, 74004) according to the manufacturer’s protocol. RNA concentration was analyzed using Nanodrop 2000 spectrophotometer (Thermo Scientific, ND-2000), and RNA quality was measured with an Agilent Bioanalyzer 2000 (Agilent Technologies). For each RNA sample, library construction was performed using the SENSE mRNA-Seq Library Prep Kit (Lexogen) according to the manufacturer’s instructions. In brief, 2 mg of total RNA was prepared from each sample and incubated with magnetic beads coated with oligodT, after which all RNAs (except for mRNAs) were removed with a washing solution provided in the kit. The library was produced by the random hybridization of heterodimers (starter/stopper) to the poly(A) RNA bound to the magnetic beads. These heterodimers contained Illumina-compatible linker sequences. Reverse transcription and ligation reaction were performed to extend the starter to the next hybridized heterodimer. High-throughput sequencing was performed as paired-end 100 sequencing using a HiSeq 2000 instrument (Illumina, USA).

### Gene set enrichment analysis

To investigate whether the two groups (e.g., siCtrl versus sir*ad51*) were significantly different, GSEA was performed using the software offered by the Broad Institute (https://gsea-msigdb.org/gsea/index.jsp). Differences in gene expression patterns between two conditions were considered significant whether most of the gene sets were in the upregulated or downregulated region of the profile. Based on the Kolmogorov-Smirnov test, GSEA calculated the enrichment score for the two conditions to measure the degree to which the level of gene expression for each of the conditions was enriched in the upregulated or downregulated region of the profile.^46^ A normalized enrichment score was calculated from the enrichment score to determine the number of significant genes.^46^

### Data analysis

The raw sequencing data were quality checked using FastQC. Adapter and low-quality reads (<Q20) were removed using the FASTX Trimmer and BBMap tools. The trimmed reads were then mapped to the reference genome using TopHat. Gene expression levels were estimated as FPKM (fragments per kb of transcript per million reads) values using Cufflinks. The FPKM values were normalized based on the quantile normalization method using edgeR in R. Data mining and graphic visualization were performed using the ExDEGA software (E-biogen).

### Statistical analysis

All data for this study were analyzed by the GraphPad Prism (version 9.0). Statistically significant differences (ns; no significant, ∗*p* < 0.05, ∗∗*p* < 0.01, ∗∗∗*p* < 0.001, and ∗∗∗∗*p* < 0.0001) between several groups were determined via paired two-tailed t test. For all figures, three biological replicates were performed. *p* values (paired two-tailed t test) were evaluated using GraphPad Prism 9.0 software. For Figures 3E and 3F, the length of DNA tail moments was calculated as follows: tail length × percentage of DNA in the tail = DNA tail moment. DNA tail moment values were calculated automatically by the CASP program as an average for the 30 single cells per condition. The black bars represent the average of three independent biological replicates. *p* values (paired two-tailed t test) were evaluated using the GraphPad Prism 9.0 software. For Figures 2D, 2F, 3I, 4E, 4F, 6A, and 6C, the intensities and focus number were quantified by NIS advanced software (Nikon). The error bars represent the mean ± SD values of three independent experiments (at least 30 nuclei per condition were counted). *p* values (paired two-tailed t test) were evaluated using the GraphPad Prism 9.0 software.

## Data and code availability

Total RNA sequencing reads are available under the following accession numbers in the NCBI Sequence Read Archive: SRX23002122, SRX23002123, and SRX23002124. All of the data that support the findings of this study are available from the corresponding author upon reasonable request.

## Supplemental information

Document S1. Figures S1‒S9

Document S2. Article plus supplemental information
