# Supplementary material for: Synergistic combination of RAD51-SCR7 improves CRISPR-Cas9 genome editing efficiency by preventing R-loop accumulation
Source: Mol Ther Nucleic Acids. 2024 Jul 17;35(3):102274. doi: 10.1016/j.omtn.2024.102274 (PMC11331969; doi:10.1016/j.omtn.2024.102274)
Supplement: Document S1. Figures S1‒S9 [file mmc1.pdf]

OMTN, Volume *35*

Supplemental information

Synergistic combination of RAD51-SCR7 improves CRISPR-Cas9 genome editing efficiency by preventing R-loop accumulation

Sun-Ji Park, Seo Jung Park, Yang Woo Kwon, and Eui-Hwan Choi

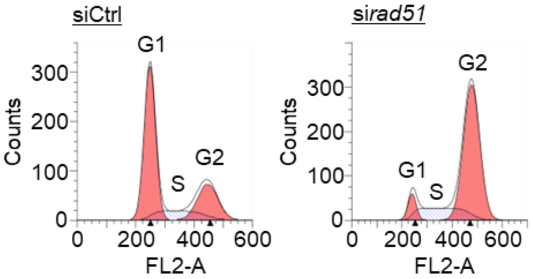

Figure S1. Cell cycle arrest by RAD51 depletion. The cell cycle profiling of HEK293T cells. Cells in each condition were analysis by flow cytometry and quantified with ModFit LT software.

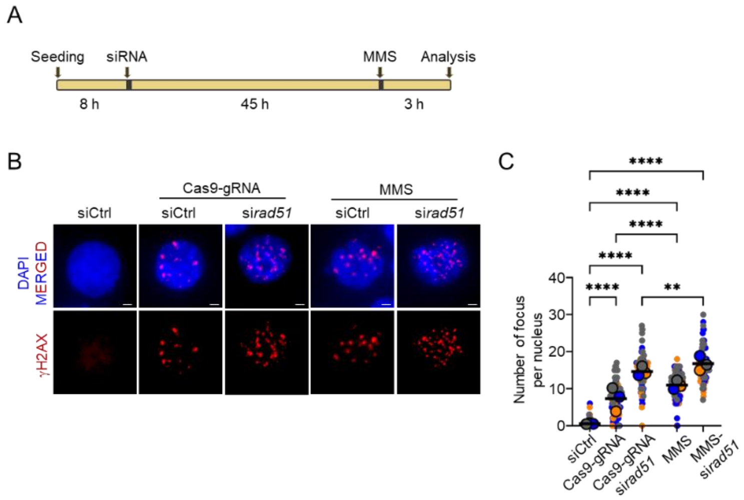

Figure S2. (A) Schematic of the siRNA and MMS treatment experiment (see Materials and Methods). (B) Representative images of γH2AX focus formation in HEK293T cells. Cells were imaged after transfection with siCtrl or si*rad51* for 48 h. Cells were transfected with Cas9-gRNA targeting hGAPDH, or treated with 1 μM MMS. Scale bars = 2.5 μm. (C) The numbers of γH2AX focus formation were quantified. The average of replicated value is represented with a larger dot, and larger bar represent the averages of three means ($n \geq 30$ for condition).

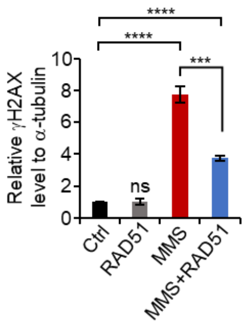

Figure S3. DNA breaks repair by expression of exogenous RAD51. Quantification of γH2AX levels. Expression levels of γH2AX in (Figure 3B) were quantified and normalized to α-tubulin (normalized values are depicted in the bar graph). Error bars indicate the mean ± SD (n= 3). Statistical significance, ns; not significant, ***P < 0.001, and ****P < 0.0001.

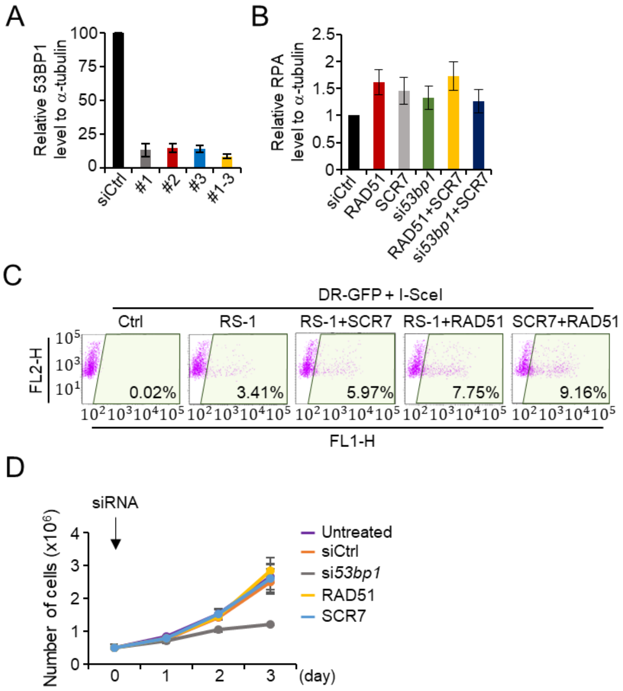

Figure S4. 53BP1 depletion and change of RPA expression level. (A) Depletion efficiency of 53BP1 in (Figure. 4A) was quantified and normalized to α-tubulin. Error bars indicate the mean ± SD (n= 3). (B) Quantitative immunoblotting analysis of relative protein levels of RPA in HEK293T cell. Protein expression was normalized to α-tubulin. Error bars indicate the mean ± SD (n= 3). (C) Quantification of HEK293T cell numbers in various conditions. siRNA and HR/NHEJ-related drugs were treated for 72 h. Error bars indicate the mean ± SD (n= 3). (D) Analysis of HR efficiency using GFP reporter-based assay. The effect of HR/NHEJ-related factors on HR pathway activation was assessed by GFP reporter-based quantification in pDR-GFP stable HEK293T cells transfected with an pCBASceI plasmid. 7.5 μM of RS-1 (Sigma, R9782) were treated into the HEK293T cell for 48 h.

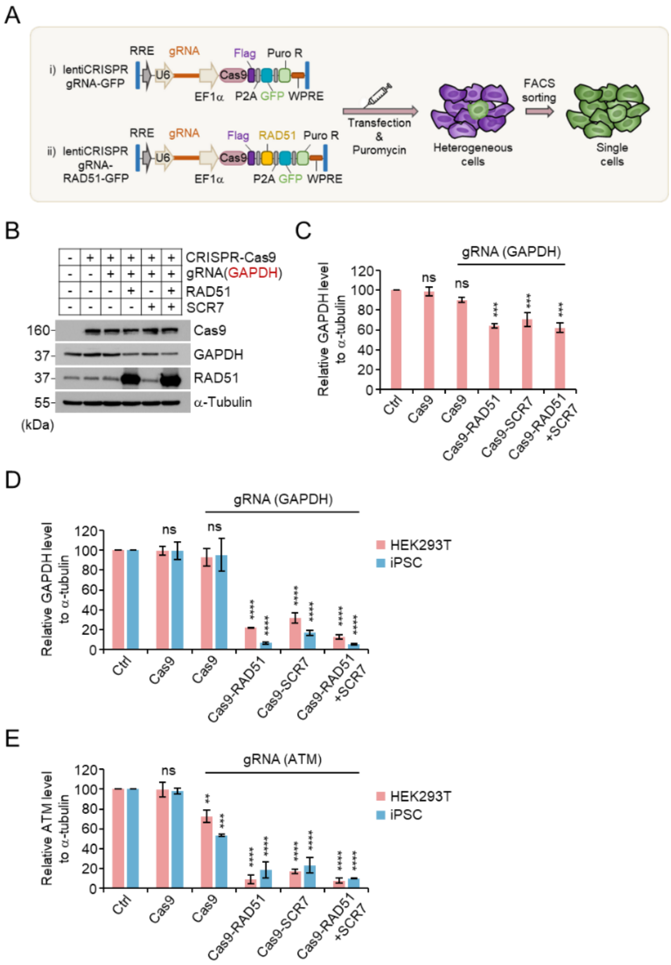

Figure S5. Diagrams of the modified all-in-one Lenti-CRISPR-Cas9 vector. (A) Specific gRNA, RAD51, and EGFP sequences were inserted into the LentiCRISPRv2 vector. Stable HEK239T cell lines expressing the all-in-one vector were generated using puromycin selection and sorted using FACS. (B) Analysis of gene editing efficiency by CRISPR-Cas9 against GAPDH in heterogenous HEK293T cells. α-tubulin used as a reference control gene for normalization. (C) The expression level of GAPDH was quantified by immunoblotting assay, with α-tubulin used as a house keeping gene for normalization. Quantification data represent the mean ± SD of 3 independent experiments. ns, not significant; ****p* < 0.001, paired two-tailed t-test. (D and E) The expression level of GAPDH or ATM was quantified by immunoblotting assay in HEK293T and hiPS stable cell lines. The level of GAPDH and ATM protein was normalized to the α-tubulin. Error bars denote the mean ± SD (n = 3). ns, not significant; ***p* < 0.01; ****p* < 0.001; *****p* < 0.0001.

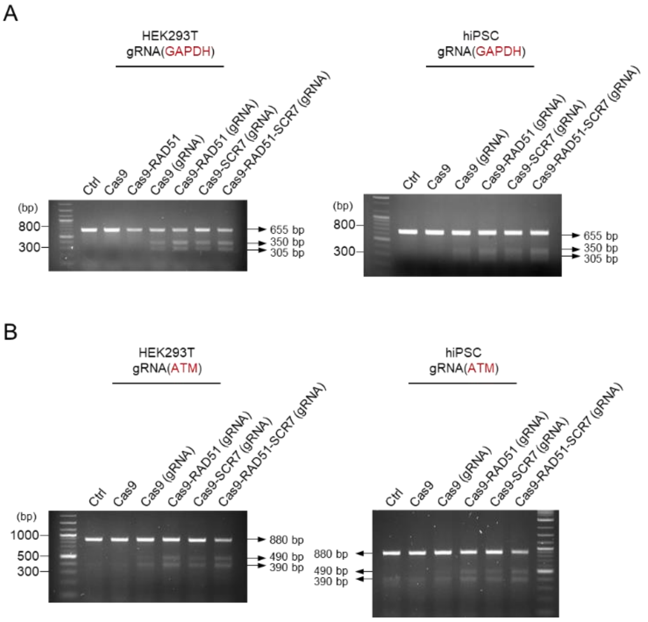

Figure S6. Analysis of genome editing efficiency by T7 endonuclease I. T7 endonuclease I analysis comparing the proportion of genomic mutation at the target site (hGAPDH and hATM) by the CRISPR-Cas9. Insertion/deletion was evaluated as the intensity ratio of the digested band to the parental band.

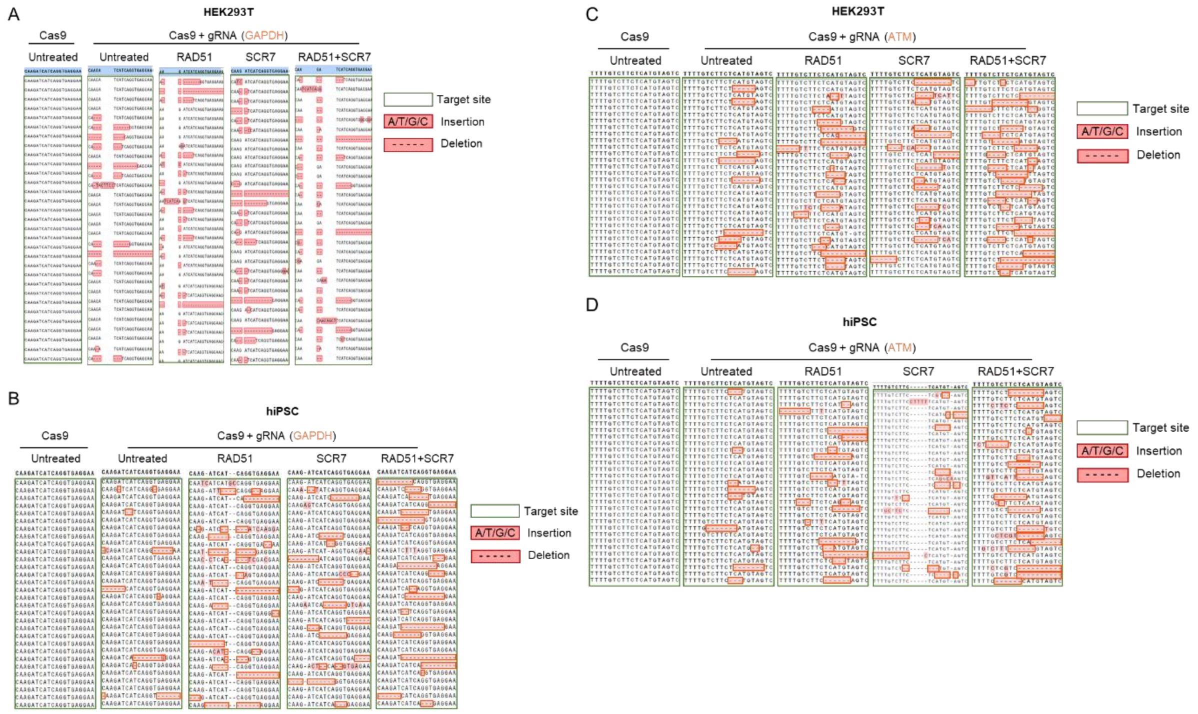

Figure S7. (A-D) Sanger sequencing for analysis of genetically modified hGAPDH and hATM in HEK293T and hiPSC. Sequencing results of a variety of mutant in CRISPR-Cas9 system.

The green box indicates gRNA(GAPDH or ATM)-targeting site. The red box mark edits including insertion, deletion.

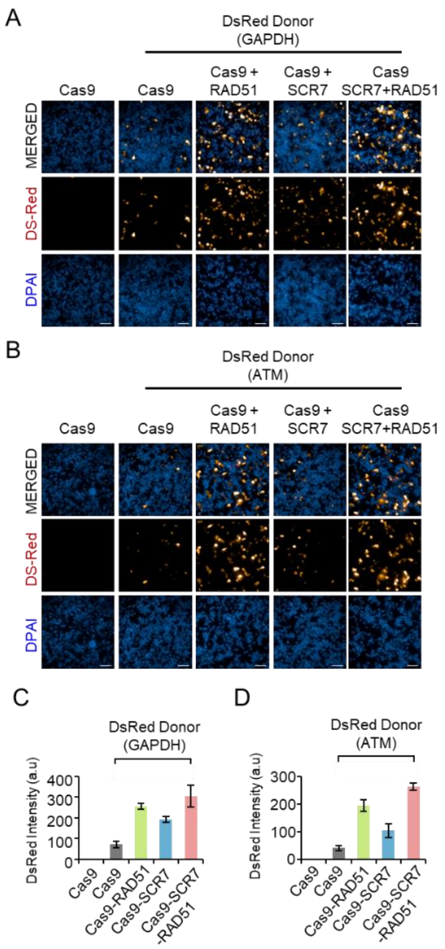

Figure S8. (A and B) Representative images showing DsRed expression in CRISPR/Cas9 system through the knock-in process. (C and D) Quantitative analysis of DsRed intensity measured in figure S8A and S8B. Error bars denote the mean ± SD (n=3).

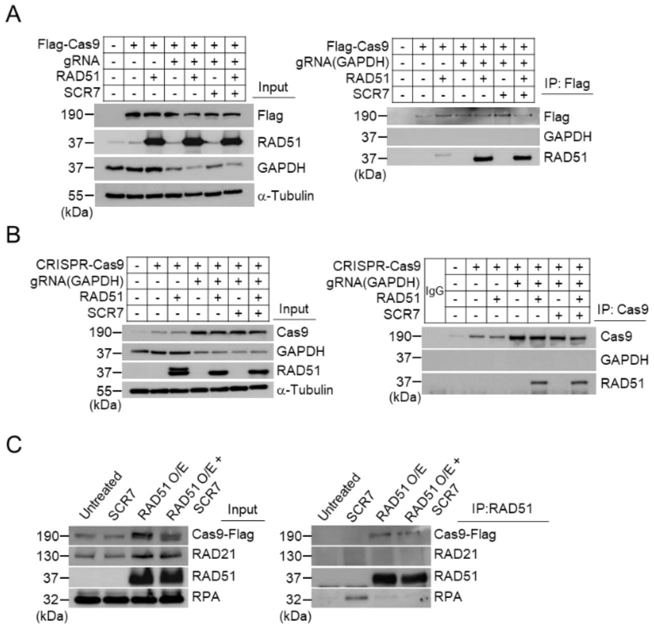

Figure S9. Interaction between RAD51 and Cas9 complex in HEK293T cells. (A-C) Immunoprecipitation (IP) analysis in HEK293T cells. RAD51 and Cas9 were pulled down the using anti-RAD51, anti-Cas9, and anti-Cas9 flag antibodies from whole-cell lysates.
